# Integrating growth and survival models for flexible estimation of size‐dependent survival in a cryptic, endangered snake

**DOI:** 10.1002/ece3.8799

**Published:** 2022-04-06

**Authors:** Jonathan P. Rose, Richard Kim, Elliot J. Schoenig, Patrick C. Lien, Brian J. Halstead

**Affiliations:** ^1^ U.S. Geological Survey Western Ecological Research Center Santa Cruz Field Station Santa Cruz California USA; ^2^ U.S. Geological Survey Western Ecological Research Center Dixon California USA

**Keywords:** capture–mark–recapture, demographic rate estimation, hierarchical model, integrated model, San Francisco gartersnake, *Thamnophis sirtalis tetrataenia*

## Abstract

Estimates of demographic rates for animal populations and individuals have many applications for ecological and conservation research. In many animals, survival is size‐dependent, but estimating the form of the size–survival relationship presents challenges. For elusive species with low recapture rates, individuals’ size will be unknown at many points in time. Integrating growth and capture–mark–recapture models in a Bayesian framework empowers researchers to impute missing size data, with uncertainty, and include size as a covariate of survival, capture probability, and presence on‐site. If there is no theoretical expectation for the shape of the size–survival relationship, spline functions can allow for fitting flexible, data‐driven estimates. We use long‐term capture–mark–recapture data from the endangered San Francisco gartersnake (*Thamnophis sirtalis tetrataenia*) to fit an integrated growth–survival model. Growth models showed that females reach longer asymptotic lengths than males and that the magnitude of sexual size dimorphism differed among populations. The capture probability and availability of San Francisco gartersnakes for capture increased with snout–vent length. The survival rate of female snakes exhibits a nonlinear relationship with snout–vent length (SVL), with survival flat between 300 mm and 550 mm SVL before decreasing for females between 550 mm and 700 mm SVL. For male snakes, survival decreased for adult males >550 mm SVL. The survival rates of the smallest and largest San Francisco gartersnakes were highly uncertain because recapture rates were very low for these sizes. By integrating growth and survival models and using penalized splines, we found support for size‐dependent survival in San Francisco gartersnakes. Our results have applications for devising management activities for this endangered subspecies, and our methods could be applied broadly to the study of size‐dependent demography among animals.

## Graphical abstract

We investigated whether San Francisco gartersnakes (*Thamnophis sirtalis tetrataenia*) exhibit size‐dependent survival by integrating growth and capture–mark–recapture models. We found that both capture probability and survival are size‐dependent in this species, with individuals of intermediate size having the highest survival rate. Integrating growth models into models of survival has the potential to reveal insights into species demography.

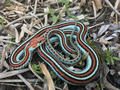

## 1 INTRODUCTION

The rates of growth, survival, and reproduction of species, and their linkage through life‐history trade‐offs, have been the subject of much empirical and theoretical research (Salguero‐Gómez et al., 2016; Stearns, 1983, 1984). In addition to interspecific variation (Shine & Charnov, 1992), vital rates vary among populations (Miller et al., 2011) and individuals (King et al., 2016; Zens & Peart, 2003). Vital rates can vary among individuals within a population depending on characteristics such as age (Gamelon et al., 2016; Zheng et al., 2007), sex (Ergon & Gardner, 2014), and size (Hyslop et al., 2012). Identifying how vital rates are influenced by individual characteristics has value for answering both basic and applied ecological questions. Interindividual variation in vital rates can reveal how selection acts to favor certain traits (Letcher & Horton, 2008) or differs among sexes (Schulte‐Hostedde et al., 2002; Toïgo & Gaillard, 2003). In wildlife conservation, quantifying variation in vital rates among individuals is valuable for designing management actions that will have the greatest effect on population growth (de Kroon et al., 2000). One trait that can explain variation in vital rates for many animals is individual body size (Sauer & Slade, 1987a).

Body size is a fundamental trait that influences metabolism (Kleiber, 1947), lifespan (Speakman, 2005), and age‐at‐maturity (Stearns, 1984). Growth (Armstrong & Brooks, 2013; King et al., 2016), survival (Rose, Wylie et al., 2018; Sauer & Slade, 1987b), and fecundity (Rose, Ersan et al., 2018; Weatherhead et al., 1995) can all be size‐dependent in animals. Researchers have developed a variety of models of individual growth (Andrews, 1982; Armstrong & Brooks, 2013; Keevil et al., 2021) and survival (Lebreton et al., 1992) to estimate these vital rates and explore how covariates explain individual, spatial, and temporal variation. Integrating growth and survival models can reveal insights into life‐history patterns that would not be identifiable from either model alone (Reinke et al., 2020). For example, estimating the effect of individual size on survival probability in a capture–mark–recapture (CMR) model requires a value for the size of each individual during each sampling period. For elusive animals with low recapture probabilities, it is impossible to measure each individual during each sampling period. If individuals cannot be measured during each sampling period, a growth model can be used to estimate size when it is unobserved (Bonner & Schwarz, 2006; Schofield & Barker, 2011). Body size can also influence capture probability (Rose, Wylie et al., 2018; Schofield & Barker, 2011) and availability for capture (i.e., emigration; Riecke et al., 2018); therefore, estimates of the size–survival relationship could be confounded unless data are analyzed in a CMR model that separates the observation, emigration, and survival processes (Kendall et al., 1997; Koons et al., 2009). Integrating a model of individual growth into a CMR model using Bayesian methods enables recapture and survival probabilities to be modeled as a function of individual size, using estimated size (with uncertainty) when no empirical data are available (Reinke et al., 2020; Rose, Wylie et al., 2018).

Determining the shape of the functional relationship between vital rates and individual covariates presents another challenge. Studies often assume a linear relationship between size and survival on the logit scale (Hansen et al., 2015; Wallace et al., 2013). There might be no reason to assume *a priori* a linear relationship between size and survival, and enforcing a linear function could lead to a misspecified model and researchers concluding any relationship is weak or nonexistent. For example, survival might increase with size up to a point before approaching an asymptote or declining for individuals above a certain size threshold (Brown & Weatherhead, 1999; Doak et al., 1994). If a linear relationship is not the only plausible option, one approach is to fit and compare multiple parametric functions (e.g., linear, quadratic, asymptotic) in a model selection framework (Rose, Wylie et al., 2018). This does not completely resolve the issue, because each parametric function enforces a particular shape on the size–survival curve, which can lead to unrealistic behavior in the tail, such as a symmetrical decrease in survival for large and small individuals with a quadratic function (Gimenez et al., 2006). Choosing a suboptimal parametric function when estimating relationships between individual covariates and vital rates can have cascading effects when vital rate functions are used to construct a demographic population model (Dahlgren et al., 2011), which could hinder such a model's ability to provide insight into management of sensitive species. Nonparametric spline functions present an appealing option when the shape of the functional relationship is unknown, because the data dictate the shape of a flexible curve (Gimenez et al., 2006). To prevent overfitting, penalized splines draw coefficients from a shared distribution, shrinking spline parameters toward zero if there is little support for their effect (Crainiceanu et al., 2005). Fitting splines can be thought of as a form of data exploration (Tredennick et al., 2021), rather than strict hypothesis testing about how vital rates vary with individual traits. In practice, spline regressions have been used to derive smooth functional relationships between vital rates and covariates in demographic models (Robinson & Wilson, 2021; Schwinn et al., 2017).

Using nonparametric splines in conjunction with CMR models has the potential to reveal valuable information on the demography of species of conservation concern. Here, we fit an integrated growth–survival model to long‐term CMR data from an endangered snake subspecies, the San Francisco gartersnake (*Thamnophis sirtalis tetrataenia*). Capture probabilities for this species are low (Halstead et al., 2011; Reeder et al., 2015); therefore, fitting a growth model was necessary to assess how individual size and sex affected survival, recapture probability, and availability for capture. We also evaluated whether and how individual growth and survival differ among populations. Our results provide the first estimates of size‐dependent survival in San Francisco gartersnakes and vital rate functions that could be used to build demographic models for this endangered subspecies. Our methods are an example of how integrating multiple demographic data types into a single model can provide greater insight into the life history of elusive species.

## 2 METHODS

### 2.1 Study species and sites

**FIGURE 1:**
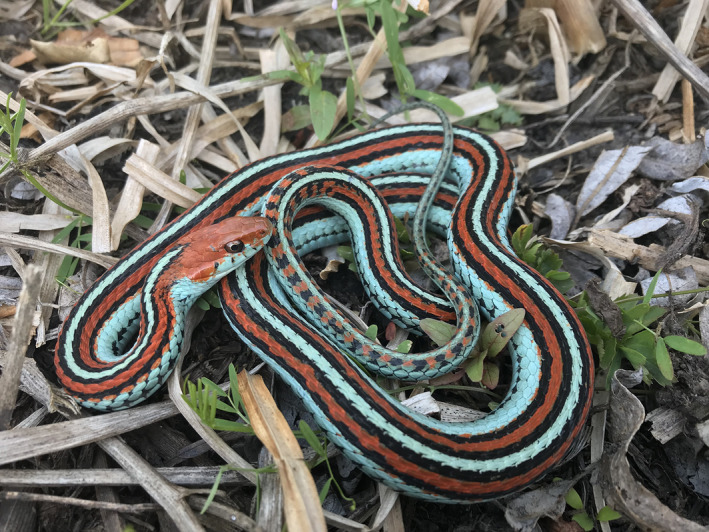
A San Francisco gartersnake (*Thamnophis sirtalis tetrataenia*) in its natural habitat in San Mateo County, California, USA. Photograph by Elliot Schoenig, U.S. Geological Survey. Public Domain

The San Francisco gartersnake (Figure 1) is a subspecies of the common gartersnake (*T*. *sirtalis*) that occurs in San Mateo and northwestern Santa Cruz counties in California, USA, and is listed as endangered under the federal Endangered Species Act (ESA, 1973; U.S. Fish & Wildlife Service, 1967) and by the state of California (Fish & Game Commission, 1971). Common gartersnakes occur in various types of habitat (Fitch, 1941), but the San Francisco gartersnake subspecies is reliant on wetlands, riparian forest, and adjacent upland habitat (Barry, 1994; U.S. Fish & Wildlife Service, 2006). San Francisco gartersnakes primarily feed on amphibians (Kim et al., 2021) including native Sierran treefrogs (*Pseudacris sierra*), California red‐legged frogs (*Rana draytonii*), and Pacific newts (*Taricha* spp.). San Francisco gartersnake populations have become more isolated as urbanization in the species’ range has altered wetlands and surrounding upland habitats (U.S. Fish & Wildlife Service, 2020; Wood et al., 2020). The minimum reported size at sexual maturity for female San Francisco gartersnakes at one population (not studied here) was 368 mm snout–vent length (SVL) with a modal SVL of 420 mm for gravid females (Reeder et al., 2015). Females are expected to mature at 2–3 years of age (U.S. Fish & Wildlife Service, 2020).

**FIGURE 2:**
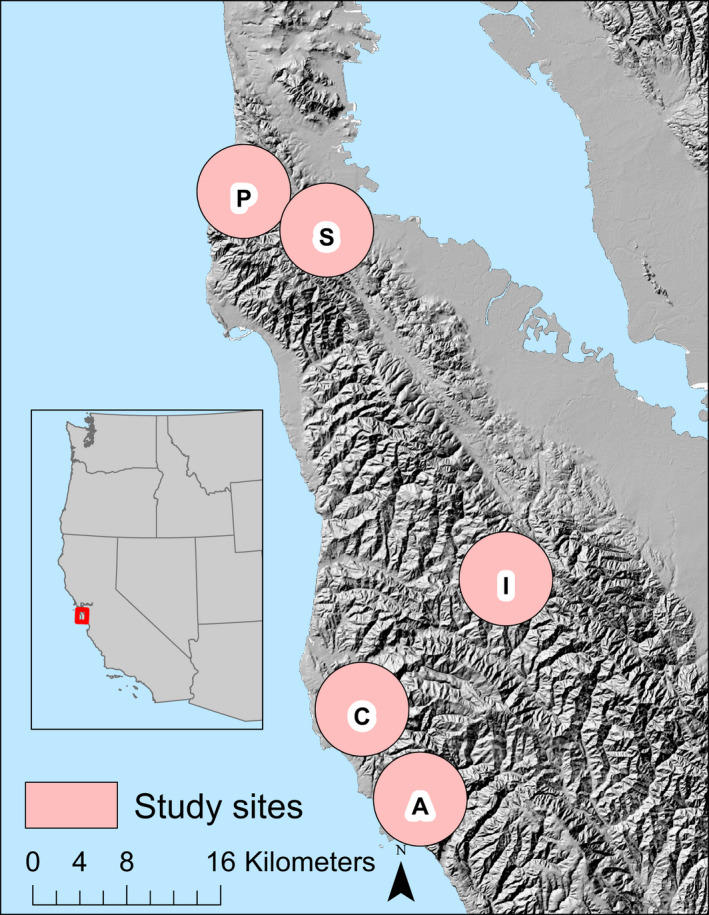
Approximate location of the five study sites for San Francisco gartersnakes (*Thamnophis sirtalis tetrataenia*) in San Mateo County, California, USA. Inset shows the location of the study region in central California. Exact locations of study sites are obscured in the interest of protecting vulnerable populations of this endangered subspecies. Sites correspond to those in Wood et al. (2020) as follows: N = Año Nuevo, C = Pescadero, I = Mindego, S = Crystal Springs and Skyline, and P = Pacifica. Base map is 30‐m resolution Hillshade created by California Department of Fish and Wildlife from U.S. Geological Survey National Elevation Dataset

We sampled for San Francisco gartersnakes at a total of five sites (Figure 2). We obscure the precise locations of these sites because illegal collection is a concern for San Francisco gartersnakes (U.S. Fish & Wildlife Service, 2006). We sampled two sites in San Mateo County, California, to collect CMR data for estimating survival (Site C and Site I). We sampled at Site C between 2007 and 2020 for an average of 51 ± 11.3 (SD) (range = 21–64) days (i.e., secondary occasions) each year and at Site I between 2014 and 2017 for 53.5 ± 7.5 (48–64) days each year. In addition to our two long‐term study sites, we sampled three additional sites (Sites N, P, and S) for San Francisco gartersnakes in 2018 and 2019 for 37.5 ± 5.5 (30–45) days each year, as part of a study on population abundance and genetic diversity (Wood et al., 2020). Sites N, P, and S are all protected from human development; sites N and P are located in coastal parks and have public access, whereas Site S is located 10 km inland and is closed to the public. We use the data from these three additional sites for modeling individual growth. Despite Site S comprising two separate clusters of trap arrays, we treat Site S as a single site based on the close proximity between our trap arrays (7.7 km), the lack of a geographical barrier to movement, and snakes from this area belonging to the same regional genetic cluster (Wood et al., 2020).

Site C was an open‐space preserve in coastal southern San Mateo County (mean elevation: 110 m, range = 50–190 m) that was off‐limits for recreation but subject to cattle grazing during the 20th century until 1998, and again from 2015 to 2020. Site C is regularly covered in coastal fog (Torregrosa et al., 2016), and the diurnal temperature ranged between 3.6 and 33°C (mean = 14.9°C, SD = 3.5) during our sampling. Aquatic habitat at this site comprised five ephemeral wetlands and two permanent water bodies. Prominent wetland vegetation included spike rush (*Eleocharis* spp.), bulrushes (*Schoenoplectus* spp.), cattails (*Typha* spp.), and willows (*Salix* spp.), which provided foraging habitat for San Francisco gartersnakes. Drought from 2012 to 2015 caused the two permanent water bodies to dry in the late summer of 2014. Upland habitat was primarily coastal scrub and grazed grassland, with Douglas‐fir (*Pseudotsuga menziesii*) forest at higher elevations. Native amphibian prey species for San Francisco gartersnakes at Site C included Pacific newts, Sierran treefrogs, and California red‐legged frogs.

Site I was an open‐space preserve in central San Mateo County (mean elevation: 480 m, range = 240–660 m) that was a private cattle ranch until 2008. Diurnal temperature at Site I ranged between 9 and 30°C (mean = 17.4°C, SD = 5.0) during our sampling. Since 2008, the site has been managed to limit public access and enhance habitat for sensitive wildlife species. Management activities at Site I include controlling vegetation by removing invasive weeds and reintroducing grazing in 2015 to keep vegetation at an early‐successional stage. Aquatic habitat at Site I comprised three ephemeral wetlands and one permanent lake. As in Site C, the permanent lake at Site I dried in the late summer of 2014 for the first time in decades. The vegetation in aquatic and upland habitat at Site I was comparable to that at Site C, with the addition of mixed evergreen forest at higher elevations. Native amphibian prey taxa at Site I were similar to Site C, but invasive American bullfrogs (*Lithobates catesbeianus*) co‐occurred up until 2015; the bullfrogs were culled by site management between 2014 and 2015 (Kim et al., 2021).

### 2.2 Data collection

Most sampling for San Francisco gartersnakes took place in April and May, although occasional surveys took place in March and later in the summer in some years (Table S1). We sampled all sites for San Francisco gartersnakes using trap arrays with 8‐ to 12‐m‐long drift fences made from tempered hardwood boards that were buried 5–7 cm in the soil and stood 40 cm high. Each drift fence had a total of four wooden box funnel traps (measuring 30 cm × 40 cm × 23 cm), with two traps placed flush against either side of the fence at each end. We installed trap arrays in upland habitat adjacent to wetlands; all trap arrays were <215 m from wetlands (Table S2). Sites I, N, S, and P were each sampled with 48 funnel traps deployed along 12 drift fences, and Site C was trapped with 96 funnel traps along 24 drift fences to cover the larger area of the site (Table 1). Trap arrays were installed at the same location each year, with minor changes if the wetland area expanded to inundate the normal location of a trap array. In addition to funnel traps, artificial cover objects were deployed at sites C and I, and snakes were captured by hand opportunistically during trap checking at all sites. The number of cover objects active each year at Site C was dependent on environmental conditions and space use of the cattle (Table S1). Funnel traps were checked daily while active, and we measured snout–vent length (SVL) to the nearest millimeter (by laying snakes on a meter stick) and mass to the nearest gram (using Pesola^®^ spring scales) of all captured snakes. We determined snake sex by cloacal probing and marked snakes by branding ventral scales with a unique code (Winne et al., 2006). We measured air temperature in the field while checking traps, and recorded whether precipitation fell in the prior 24 h. We acquired data on mean daily relative humidity from interpolated gridMET data (Abatzoglou, 2013). For some dates when air temperature was not collected in the field, we used gridMET data to impute the air temperature, based on a linear regression model relating air temperature recorded in the field and gridMET mean daily temperatures for that site (Appendix S1).

**TABLE 1:** Summary of trapping effort, number of individual San Francisco gartersnakes (*Thamnophis sirtalis tetrataenia*) captured, and sex of captured individuals for each of five study sites. Each trap array comprises four funnel traps, and the same number of trap arrays was used for each year of sampling at a site

Site	Sampling period	Trap arrays	Region	Individuals captured	Female	Male	Unknown	Individuals with growth data
N	2018–2019	12	Coastal	85	37	46	2	20
C	2007–2020	24	Coastal	678	303	355	20	90
S	2018–2019	12	Inland	91	55	33	3	8
I	2014–2017	12	Inland	207	87	114	6	41
P	2018–2019	12	Coastal	31	20	11	0	4

### 2.3 Growth model

We fit a von Bertalanffy (VB) growth model based on Armstrong and Brooks (2013) to snake SVL data from recaptures. Unlike Armstrong and Brooks (2013), we did not incorporate biphasic growth, because preliminary model fitting indicated there was no evidence for a fixed change in growth rates as snakes increased beyond a particular size threshold. This growth model takes the general form (Equation 1)

(1) $${\mathrm{EL}}_{i,t}=a_{i}‐\left(a_{i}‐L_{i,t‐1}\right)\exp\left[‐\frac{k_{i,t}}{a_{i}}\left(\Delta t\right)\right]$$

where *EL_i_*,*_t_* is the expected SVL of snake *i* at time *t*, *a_i_* is the asymptotic SVL of snake *i*, *k_i_*_,_ *_t_* is the somatic growth rate of snake *i* at time *t*, andΔt is the number of days between captures, including the inactive season. Although the exponential component of the VB growth model is usually parameterized in terms of *k* (Wang, 1998), we followed Armstrong and Brooks (2013) and substituted *k*/*a* for *k*, which allows for individual variation in growth rate to be independent from individual variation in asymptotic size. The observed SVL (*L_i_*_,_ *_t_*) is drawn from a normal distribution centered on *EL_i_*_,_ *_t_* with random variation around the expected size at time *t* (*ε_t_*) that encompasses both measurement error and individual variation around expected size (Equation 2).

(2) $$L_{i,t}∼N\left({\mathrm{EL}}_{i,t},\varepsilon_{t}\right)$$

We modeled log(*k*) as a function of sex (fixed effect) and random effects of individual (*i*), site (*s*), and year (*t*) (Equation 3).

(3) $$log\left(k_{i,t}\right)=\;\mu_{f}+{\beta_{m}*m_{i}+\alpha }_{sex,s}+\zeta_{t}+\iota_{i}$$

The variable *µ_f_* is the mean value of *k* for a female snake, *β_m_* is the effect of maleness on *k*, *m_i_* is a binary indicator of whether an individual is male (1) or not (0), and *α_sex_*_,_ *_s_*, *ζ_t_*, and *ι_i_* are site (by sex), year, and individual random effects, respectively.

We modeled asymptotic length (*a_i_*) as a function of sex and site (Equation 4). The mean estimate of *a* for a female snake is *υ_f_*, *θ_m_* is the effect maleness on *a*, *m_i_* is the binary male indicator defined above, and *η_sex_*,*_s_* is the sex‐ and site‐specific random effect on *a*.

(4) $$a_{i}=\;\upsilon_{f}+\;\theta_{m}*m_{i}+\eta_{sex,s}$$

We defined the site, year, and individual random effects with normal distributions with a mean of zero and standard deviation shared among members of that cluster level (Table 2). The site random effects on *k* and *a* were each sex‐specific, which allowed these growth parameters to vary among sites in different ways for females and males, because interpopulation differences in sexual size dimorphism have been found in other subspecies of *T*. *sirtalis* (Krause et al., 2003). We then used the estimated *a* and *k* parameters and Equation 1 to project size at age for San Francisco gartersnakes using a neonate size at birth of 165 mm SVL based on measurements reported by Barry (1994).

**TABLE 2:** Parameters, prior distributions, and summary of posterior distributions from the von Bertalanffy growth model for San Francisco gartersnakes (*Thamnophis sirtalis tetrataenia*) captured at five sites from 2007 to 2020. The prior “*N*(mean, SD)” represents a normal distribution with mean and standard deviation (SD), “Exp(scale)” is an exponential distribution with a scale parameter, and “–” indicates a derived parameter. Mean and SD are summary statistics from the posterior distribution for each parameter, and 2.50% and 97.50% indicate percentiles of the posterior distribution

Parameter	Description	Prior	Posterior			
			Mean	SD	2.50%	97.50%
*µ_a_*_,f_	Mean asymptotic length, females	*N*(750,200)	737.23	22.67	693.79	783.25
*µ_a_*_,m_	Mean asymptotic length, males	–	550.66	17.50	517.72	588.16
β2_m_	Male effect on asymptotic length	*N*(0,200)	−186.57	28.38	−242.10	−129.37
*µ_k_*_,f_	Mean log growth coefficient, females	*N*(0,10)	5.55	0.34	4.80	6.16
*µ_k_*_,m_	Mean log growth coefficient, males	–	5.90	0.40	5.02	6.62
β1_m_	Male effect on log(*k*)	*N*(0,10)	0.34	0.50	−0.67	1.34
*σ_k_*_,_ *_t_*	Standard deviation of year random effect on log(*k*)	Exp(1)	0.14	0.09	0.01	0.33
*σ_k_*_,_ *_s_*	Standard deviation of site random effect on log(*k*)	Exp(1)	0.56	0.31	0.09	1.30
*σ_k_*_,_ *_i_*	Standard deviation of individual random effect on log(*k*)	Exp(1)	0.42	0.06	0.30	0.56
*σ_a_*_,s_	Standard deviation of site random effect on *a*	Exp(0.1)	25.70	9.83	10.92	48.92
*σ_e_*	Standard deviation in residual variation of individual length	Exp(0.1)	23.33	2.10	19.51	27.73

We implemented the VB growth model using Markov chain Monte Carlo (MCMC) sampling in JAGS (Plummer, 2003) accessed through R version 4.0.5 (R Core Team, 2021) using the “runjags” package (Denwood, 2016). We ran the VB growth model on four chains for 2,500,000 sampling iterations each, after discarding the initial 20,000 iterations as burn‐in. The resulting output was thinned by a factor of 100, resulting in a final sample of 100,000 model iterations used for inference. We evaluated model convergence by inspecting MCMC trace plots and calculating the Brooks–Gelman–Rubin statistic ($\hat{R}$; Brooks & Gelman, 1998). All parameters had $\hat{R}$ < 1.01, and chains showed no evidence of lack of convergence. We tested the goodness of fit of the VB growth model using a posterior predictive check, by comparing the sum of squares of residuals for observed snake SVLs at recaptures compared with expected values, and repeating this calculation of residuals for replicate snake SVLs generated from the model (Keevil et al., 2021). There was no evidence of lack of fit in the scatterplot of residual values (Figure S1, Appendix S1), and the Bayesian *p*‐value was .48, indicating adequate fit of the model to growth data (Kéry & Schaub, 2012). The connection between parameters in equations (1), (2), (3), (4) above and JAGS growth model code is presented in Table S3.

### 2.4 Robust‐design model

We fit a hierarchical, multistate, robust‐design model (Riecke et al., 2018) to our CMR data of San Francisco gartersnakes from two long‐term study sites (Site I and Site C) to estimate the annual apparent survival (*ϕ*), probability of being available for capture (*γ*), and daily recapture probability (*p*) at each site, while accounting for variation due to individual size. Our robust‐design model conditioned on initial capture and is a multistate version of a Cormack–Jolly–Seber model (Cormack, 1964; Jolly, 1965; Seber, 1965). Only snakes first captured at least one year prior to the final year of sampling at each site were included in the robust‐design model. To assess how individual size (SVL) and sex of San Francisco gartersnakes affected survival, capture, and availability, we used Bayesian imputation by integrating the VB growth model into the robust‐design model. This allowed us to infer the size of snakes (with uncertainty) when they were not captured, or on the few occasions (~1% of captures) when snakes were not measured upon capture. For eight snakes (out of 745) that were not measured during their first capture, their initial SVL was imputed based on a normal distribution with the mean and standard deviation of SVL for that sex. We set informative priors for the VB growth parameters in the robust‐design CMR based on the posterior distributions from the VB growth model (Table 2), with slightly wider uncertainty.

Under the robust design, sampling consists of widely separated primary periods (*t*) each composed of multiple secondary periods (*j*) separated by small temporal intervals; populations are assumed closed between secondary periods (i.e., no mortality or migration), while populations are assumed to be open (i.e., mortality and migration can occur) during the larger intervals between primary periods (Pollock, 1982). We assumed that individual snakes are in one of three states during each primary period: available for capture, temporarily unavailable, or permanently unavailable (dead or permanent emigrant). We used a random emigration formulation of the robust‐design model that assumed availability for capture in one primary period did not depend on an individual's state in the previous primary period. We assumed random emigration because our sampling was focused near aquatic habitats where San Francisco gartersnakes are likely to forage for amphibian prey each spring (Kim et al., 2021), and therefore, we did not expect an individual's availability for capture in one year to influence their availability the next year. We created our robust‐design survival model in a Bayesian framework following the parameterization of Riecke et al. (2018), which allows for unbiased estimates of apparent survival (*ϕ*), availability (*γ*), and capture probability (*p*). The key innovation in the model of Riecke et al. (2018) is that data likelihoods are modified such that only individuals known to be available for capture in a given secondary period affect the capture probability for that secondary period.

We included potential effects of snake SVL on *ϕ* and *p* by fitting penalized splines (Eqs. 5,6). Given the difficulty of obtaining precise estimates of availability on‐site for capture without auxiliary data (Bird et al., 2014), and based on preliminary model fitting in which a spline function for *γ* approximated a linear relationship with SVL, we fit a simple linear effect of SVL on availability (Equation 7). In equation 5, π*_ϕ_*_,_ *_sex_*_,_ *_s_* is the sex‐ and site‐specific intercept of apparent survival on the logit scale, and year‐specific random effect on *ϕ* is *ξ* *_t_*. In equation 6, ο*_p_*_,_ *_sex_*_,_ *_s_* is the sex‐ and site‐specific intercept of *p* on the logit scale, and *ω_s_*_,_ *_t_* is the site and year random effect on *p*. In addition to the effect of snake size on *p*, we included potential linear effects of air temperature, recent precipitation (rain the last 24 h before checking traps), relative humidity, and day of year on *p*, where the covariate matrix is ***W***, and the vector of coefficients is *δ_p_* (Equation 6). In equation 7, ς_γ_ *_sex_*_,s_ is the sex‐ and site‐specific intercept of availability on the logit scale, *λ_γ_* is the slope of the relationship between snake SVL and availability for capture, and ν *_s_*_,_ *_t_* is the site and year random effect on *γ*. We constrained the effects of survey covariates (e.g., air temperature, day of year) on capture probability to be linear functions because our two‐month sampling period did not cover the full seasonal variation in weather at our study sites. Although we believe that using linear functions was a reasonable assumption for this short time period, it is likely that some of these covariates might have more complex relationships with capture probability when evaluated over a wider range of values (e.g., capture probability might be highest for an optimal temperature range and decline temperatures above and below that range).

(5) $${logit(\phi }_{i,t})\;={\pi_{\phi ,sex,s}+\;f}_{\phi }\left(x_{i,t}\right)+\xi_{t}$$

(6) $$logit\left(p_{i,t,j}\right)\;={o_{p,sex,s}+\delta_{p}W+\;f}_{p}\left(x_{i,t}\right)+\omega_{s,t,j}$$

(7) $${logit(\gamma }_{i,t})\;=\varsigma_{\gamma ,sex,s}+\lambda_{\gamma }x_{i,t}+\nu_{s,t}$$

We fit low‐rank thin plate splines following the methods of Crainiceanu et al. (2005). We divided the range of SVL values into *G* = 5 knots (where *g* indicates an individual knot) and fit spline functions (degree = 3) to each knot (Equation 8), where *q* is a fixed effect of SVL, *b_g_* are random coefficients, *κ* are fixed knots along the range of observed SVL values, *κ_g_* is the quantile of SVLs that matches *g*/*G* + 1, and (*x*–*κ_g_*)_+_ represents max(0, *x*–*κ_g_*), and *d* is the degree of the spline. The splines are penalized because each *b* is drawn from a normal distribution (*N*) with a shared standard deviation parameter (*σ_b_*), making each *b* parameter analogous to a random effect (Equation 9). The penalty that comes from drawing splines from a shared distribution shrinks the *b* parameters toward zero and limits the smooth function from assuming too complex a shape.

(8) $${f(x}_{i,t})=q*x_{i,t}+\;\sum_{g=1}^{G}b_{g}{\left(x‐\;\kappa_{g}\right)}_{+}^{d}$$

(9) $${b_{g}(x}_{i,t})\;∼\;N\left(0,\sigma_{b}\right)$$

For a given response variable (*ϕ* or *p*), we used a shared spline function, *f*(*x*) among sites and both sexes. In other words, although the intercept could differ among sexes and sites, we assumed that the size–survival function would be the same for each site‐sex group, likewise for the size–capture probability function. We also assumed a shared slope among sites and sexes for the size–availability functions. We made the assumption of shared shape of size effects among sites because most of our CMR data came from Site C, and we lacked sufficient data to estimate independent functions for snakes from Site I. We tested for potential correlation among annual growth and survival rates by using a covariance matrix to define annual random effects for these parameters (Appendix S1). Because a small number of snakes were of unknown sex, we imputed the sex of these snakes using a Bernoulli prior for a binary indicator of male sex with a probability of 0.5. We assumed a fixed 1:1 sex ratio for snakes of unknown sex based on previous estimates of the sex ratio in our study populations (Wood et al., 2020).

We ran the robust‐design model using Markov chain Monte Carlo (MCMC) sampling in JAGS (Plummer, 2003) accessed through R version 4.0.5 (R Core Team, 2021) using the “runjags” package (Denwood, 2016). We ran the robust‐design model on 10 chains for 50,000 iterations per chain after discarding a burn‐in of 10,000 iterations. We thinned the resulting chains by a factor of 10, resulting in a final MCMC sample of 50,000 iterations. As for the VB growth model, we evaluated model convergence by inspecting trace plots and calculating $\hat{R}$; all $\hat{R}$ were ≤1.05, and chains showed good mixing. We also evaluated the goodness of fit of the robust‐design model by simulating replicate capture data from the model and comparing how replicate data and real data deviated from expected values of the number of recaptures (Rose, Wylie et al., 2018). We used the Freeman–Tukey statistic as a measure of the discrepancy between expected values and real and simulated data (Brooks et al., 2000). We made a scatter plot of the deviation for replicate and real data and calculated a Bayesian *p*‐value to determine whether the robust‐design model provided adequate fit to the data (Figure S2). The Bayesian *p*‐value for the robust‐design model was 0.48, indicating adequate fit to the data. The connection between parameters in equations (5), (6), (7), (8), (9) above and JAGS CMR model code is presented in Table S3. Model code is available at Rose et al. (2022a) (https://doi.org/10.5066/P9700BBK), and data to reproduce occupancy analyses area are available at Rose et al. (2022b; https://doi.org/10.5066/P9SC36I8).

## 3 RESULTS

### 3.1 Growth model

We captured 1090 individual San Francisco gartersnakes at the five study sites. The largest captured male measured 635 mm SVL, and the largest female measured 825 mm SVL. Of these 1090 San Francisco gartersnakes, 163 individuals (72 females, 89 males, and 2 of unknown sex) were captured and measured more than once, and data from these snakes were used to estimate San Francisco gartersnake growth. Most of the growth data came from Site C (*n* = 91 individuals), followed by Site I (41), Site N (20), Site S (7), and Site P (4). Of the 163 San Francisco gartersnakes with growth data, 148 were caught twice and 15 were caught three times, resulting in 178 growth increments. The average interval between captures was 507.0 days (SD = 284.7), and the majority of growth measurements (126/178) were taken in consecutive years, with a further 41 growth increments separated by 2 years, and 11 increments separated by 3 or more years (max = 6 years). Most recaptured snakes (154/163) were >300 mm SVL at first capture, so minimal data were available to estimate growth of neonates.

**FIGURE 3:**
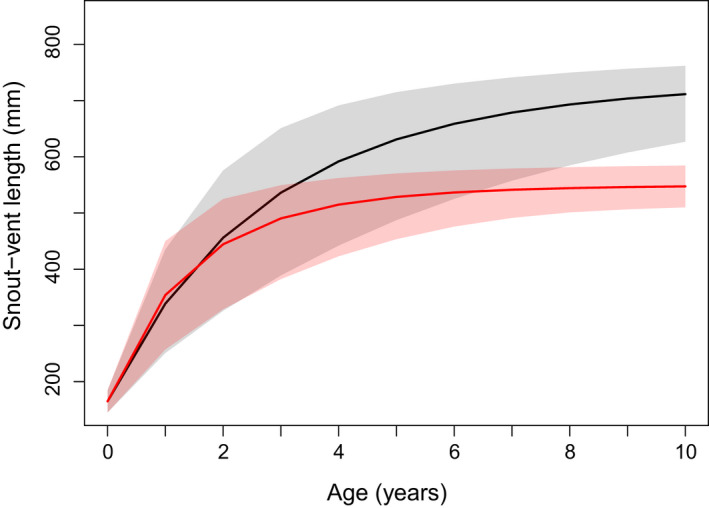
Growth trajectory for female (black line, gray shading) and male (red line, red shading) San Francisco gartersnakes (*Thamophis sirtalis tetrataenia*) as a function of age. Each trajectory is based on the mean parameter estimates from a von Bertalanffy growth model (i.e., the values for an “average” site). The solid line represents the median projected size at each age, and the shaded region is the 95% credible interval. Growth projections assume that neonates of both sexes have mean SVL = 165 mm (SD = 10 mm) based on measurements reported in Barry (1994)

Female San Francisco gartersnakes reached greater asymptotic SVL than males (Table 2, Figure 3). There was weak support for male San Francisco gartersnakes approaching their asymptotic SVL faster than females on average (posterior probability [*k*_m_ > *k*_f_] = 0.80; Table 2). Estimates of *k* were similar between males and females for each site except Site C, where *k*_m,C_ had 0.96 posterior probability of being greater than *k*_f,C_ (Figure S3). Individuals of both sexes grew at similar rates until reaching 2 years of age, at which point the growth trajectories diverged, with male SVL rapidly approaching its asymptote by ages 4–5 years, and female SVL continuing to increase before beginning to plateau around 7–8 years (Figure 3). There was more variation in male asymptotic SVL among sites than female asymptotic length; in particular, male asymptotic SVL was greater at Site I than at Site C (Figure S4). Greater variance in *k* was explained by differences among sites and individuals than differences among years (Table 2).

### 3.2 Robust‐design analysis of CMR data

The multistate robust‐design model was fit to CMR data from 745 snakes (333 females, 387 males, and 25 unknown sex) captured at Site C and Site I. At Site C, 603 snakes (272 females, 311 males, and 20 unknown) were captured a total of 829 times (including multiple captures within the same year). Of the 603 snakes captured at Site C, 510 were captured in only one year, 84 were captured in two years, and 9 were captured in 3 years. At Site I, 142 snakes (61 females, 76 males, and 5 unknown) were captured a total of 262 times. Of the 142 snakes captured at Site I, 101 were captured in only one year, 35 were captured in two years, and 6 were captured in 3 years.

**FIGURE 4:**
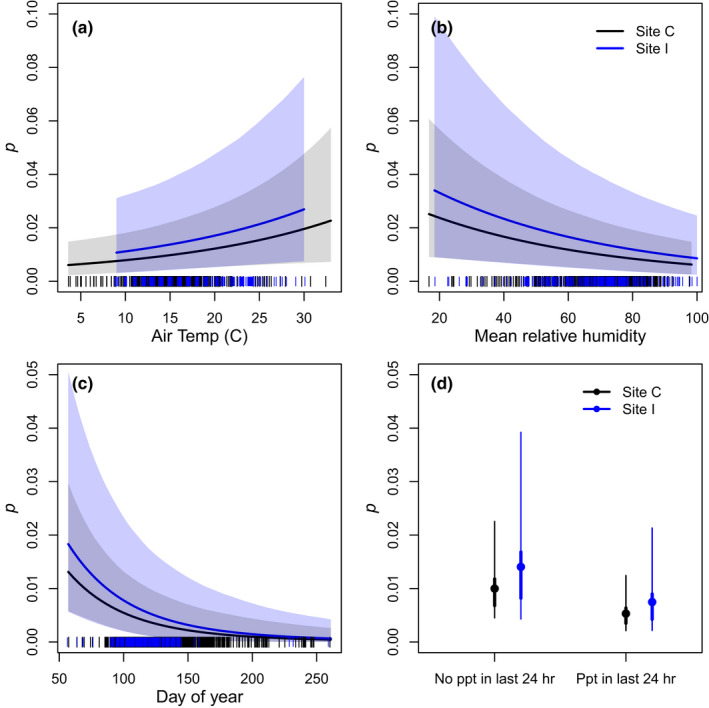
Relationship between environmental (air temperature, relative humidity, and precipitation) and seasonal (day of year) covariates and daily recapture probability, *p*, for San Francisco gartersnakes (*Thamophis sirtalis tetrataenia*). In panels a–c, lines represent mean posterior predictions, and shaded regions are symmetrical 95% credible intervals. In panel d, circles represent posterior means, thick lines represent 50% credible intervals, and thin lines represent 95% credible intervals. These relationships are based on data from female snakes, but results for male snakes were qualitatively similar. Vertical dashes on the *x*‐axis represent observed covariate values at Site C (black) and Site I (blue), respectively. Air temperature is in degrees Celsius; ppt = precipitation

**FIGURE 5:**
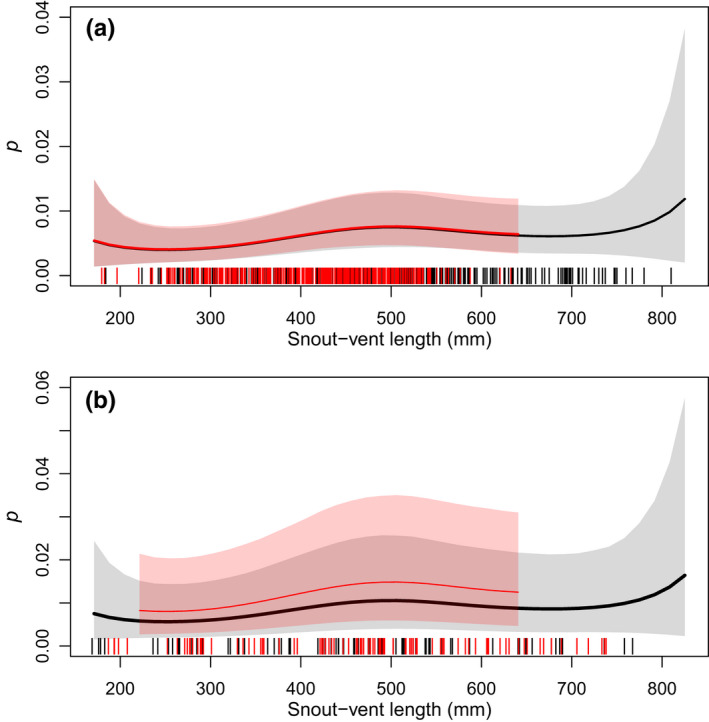
Estimated relationship between snake snout–vent length (SVL) in mm and *p*, the daily recapture probability of San Francisco gartersnakes (*Thamnophis sirtalis tetrataenia*), from the robust‐design Cormack–Jolly–Seber model. Panel a presents estimated relationships from Site C, and panel b presents estimated relationships from Site I. Solid lines are posterior medians, and shaded regions are 95% credible intervals. Black lines and gray shading represent female snakes, and red lines and shading represent male snakes. Vertical tick marks on the *x*‐axis represent the SVL of captured snakes, shifted slightly to improve visibility

Daily recapture probability (*p*) was positively related to air temperature, and negatively related to relative humidity and day of year. Capture probability was lower if there was precipitation in the 24 h before trap‐checking than if there was no precipitation (Figure 4). Mean daily recapture probability was higher at Site I than at Site C for male snakes (Pr[*p*_m,I_ > *p*_m,C_] = 0.91), but the difference between female snakes at the two sites was smaller (Pr[*p*_f,I_ > *p*_f,C_] = 0.72). At Site I, mean *p* was higher for male snakes than for female snakes (Pr[*p*_m,I_ > *p*_f,I_] = 0.96); at Site C, the two sexes had nearly equal mean values for *p* (Pr[*p*_m,C_ > *p*_f,C_] = 0.55; Table 3). Daily recapture probability was positively related to snake SVL up to approximately 500 mm SVL, after which *p* plateaued with increasing SVL (Figure 5). The probability of a snake of average length (mean = 463 mm SVL across sites) being recaptured at least once in a year (p*), given average survey conditions and 52 days of sampling (the average survey period per year), was 0.48 (0.20–0.88) and 0.59 (0.29–0.94) for female and male snakes, respectively, at Site I, and 0.39 (0.21–0.69) for female and 0.40 (0.23–0.68) male snakes at Site C.

**TABLE 3:** Parameters, prior distributions, and summary of posterior distributions from the multistate robust‐design model for San Francisco gartersnakes (*Thamnophis sirtalis tetrataenia*) captured at two sites from 2007 to 2020. The prior “*N*(mean, SD)” represents a normal distribution with mean and standard deviation (SD), “Exp(scale)” is an exponential distribution with a scale parameter, a “Beta(shape1, shape2)” is a beta distribution with two shape parameters, and Uniform(lower, upper) is a uniform distribution between a lower and upper limit. Mean and SD are summary statistics from the posterior distribution for each parameter, and 2.50% and 97.50% indicate percentiles of the posterior distribution

Parameter	Description	Prior	Posterior			
			Mean	SD	2.50%	97.50%
*p*_f,C_	Mean daily recapture probability of females at Site C	Beta(1,1)	0.010	0.005	0.005	0.023
*p*_m,C_	Mean daily recapture probability of males at Site C	Beta(1,1)	0.010	0.004	0.005	0.022
*p*_f,I_	Mean daily recapture probability of females at Site I	Beta(1,1)	0.014	0.009	0.004	0.039
*p*_m,I_	Mean daily recapture probability of males at Site I	Beta(1,1)	0.020	0.012	0.007	0.052
*α_p_*_,at_	Slope of relationship between *p* and air temperature	*N*(0,1)	0.183	0.082	0.023	0.344
*α_p_*_,rh_	Slope of relationship between *p* and relative humidity	*N*(0,1)	−0.214	0.068	−0.347	−0.080
*α_p_*_,at_	Slope of relationship between *p* and day of year	*N*(0,1)	−0.265	0.089	−0.442	−0.093
*α_p_*_,at_	Effect of precipitation in previous 24 h on *p*	*N*(0,1)	−0.652	0.194	−1.043	−0.283
*ϕ*_f,C_	Mean annual survival of females at Site C	Beta(1,1)	0.660	0.109	0.442	0.862
*ϕ*_m,C_	Mean annual survival of males at Site C	Beta(1,1)	0.521	0.098	0.334	0.720
*ϕ*_f,I_	Mean annual survival of females at Site I	Beta(1,1)	0.523	0.150	0.244	0.821
*ϕ*_m,I_	Mean annual survival of males at Site I	Beta(1,1)	0.677	0.136	0.398	0.925
*γ*_f,C_	Mean availability of females at Site C	Beta(1,1)	0.330	0.168	0.090	0.730
*γ*_m,C_	Mean availability of males at Site C	Beta(1,1)	0.651	0.145	0.365	0.915
*γ*_f,I_	Mean availability of females at Site I	Beta(1,1)	0.633	0.228	0.176	0.982
*γ*_m,I_	Mean availability of males at Site I	Beta(1,1)	0.594	0.210	0.192	0.960
*σ_p_*, *_t_*	Standard deviation of annual variation in *p*	Exp(1)	0.647	0.202	0.341	1.143
*σ_ϕ,t_*	Standard deviation of annual variation in *ϕ*	Exp(1)	1.186	0.483	0.431	2.323
*σ_γ,t_*	Standard deviation of annual variation in *γ*	Exp(1)	1.359	0.636	0.193	2.787
*ρ_k_*, *_ϕ_*	Correlation between annual random effect on *k* and *ϕ*	Uniform(−1,1)	0.012	0.503	−0.908	0.883

**FIGURE 6:**
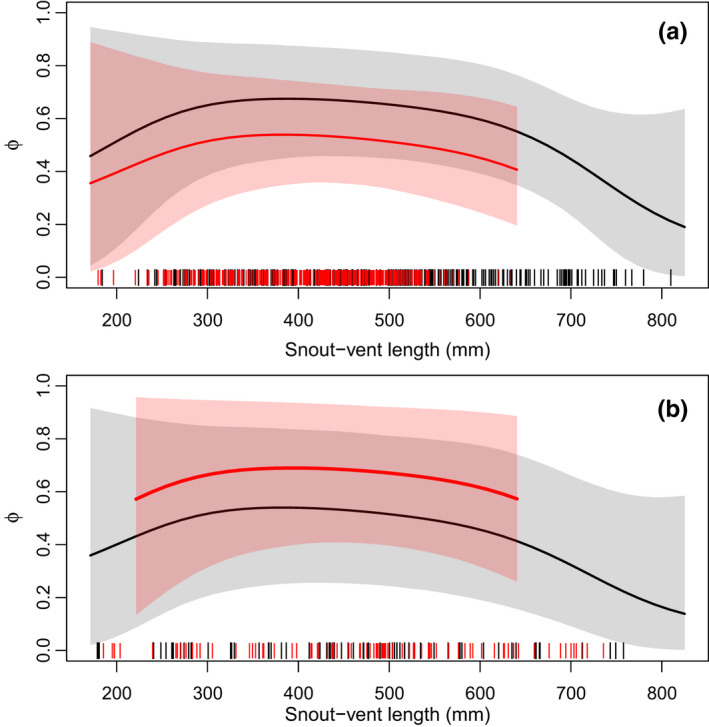
Estimated relationship between snake SVL and *ϕ*, the annual apparent survival probability for San Francisco gartersnakes (*Thamnophis sirtalis tetrataenia*), from the robust‐design Cormack–Jolly–Seber model. Panel a presents estimated relationships from Site C, and panel b presents estimated relationships from Site I. Solid lines are posterior means, and shaded regions are 95% credible intervals. Black lines and gray shading represent female snakes, and red lines and shading represent male snakes. Vertical tick marks on the *x*‐axis represent the SVL of captured snakes of each sex, shifted slightly to improve visibility

Annual apparent survival (*ϕ*) at Site C exhibited a flat relationship with snake SVL for individuals between 300 mm to 550 mm, followed by a decline in survival for snakes between 550 mm and 700 mm SVL (Figure 6). Below 300 mm SVL and above 700 mm SVL, there were fewer recaptures of marked individuals at Site C and *ϕ* was highly uncertain (95% CI width >0.50). At Site I, patterns for the posterior mean were similar, but sample sizes were smaller and uncertainty in *ϕ* was greater for all sizes. Mean annual survival for a snake of average SVL (463 mm) was higher for females than for males at Site C (Pr[*ϕ*_f,C_ > *ϕ*_m,C_] = 0.96; Table 3) but not at Site I, where there was some support for males having higher survival than females (Pr[*ϕ*_f,I_ > *ϕ*_m,I_] = 0.16; Table 3). Mean annual survival at Site C for a female snake with average SVL was 0.66 (95% CRI = 0.44–0.86) and for a male snake of the same SVL annual survival was 0.52 (0.33–0.72). At Site I, mean annual survival of a female of average size was 0.52 (0.24–0.82) and a male of average size was 0.68 (0.40–0.92). Survival fluctuated between years, with the highest survival at Site C estimated for 2008–2009 and 2009–2010 and the lowest survival from 2012 to 2013 and 2018 to 2019 (Figure S5). For the shorter time series at Site I, mean survival appeared slightly lower from 2016 to 2017 than from 2014 to 2015 and 2015 to 2016 (Figure S6). The model estimated no correlation between yearly random effects on growth (*k*) and survival within our sample (*ρ* = 0.01, −0.91–0.88).

**FIGURE 7:**
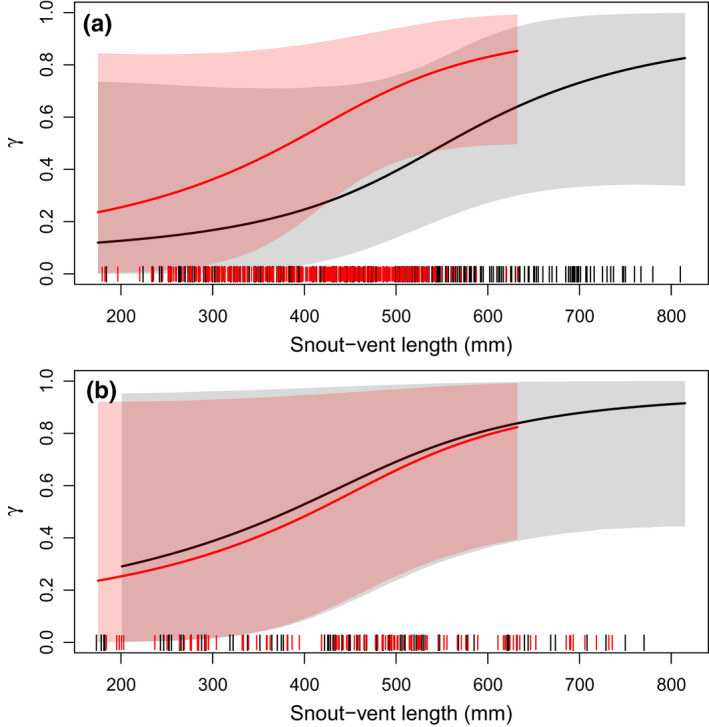
Estimated relationship between snake snout–vent length (SVL; mm) and availability for capture (*γ*) of San Francisco gartersnakes (*Thamnophis sirtalis tetrataenia*) from the robust‐design model. Panel a presents estimated relationships from Site C, and panel b presents estimated relationships from Site I. Solid lines are posterior means, and shaded regions are 95% credible intervals. Black lines and gray shading represent female snakes, and red lines and shading represent male snakes. Vertical tick marks on the *x*‐axis represent the SVL of captured snakes of each sex, shifted slightly to improve visibility

Availability for capture (*γ*) increased linearly with snake SVL (Pr[*λ_γ_* > 0] = 0.95; Figure 7). Availability for capture could not be precisely estimated for snakes <400 mm SVL. Male snakes of average length had higher average probability of being available for capture at Site C (*γ*_m,C_ = 0.65, 0.36–0.92) than females (*γ*_f,C_ = 0.33, 0.09–0.73; Pr[*γ*_m,C_ > *γ*_f,C_] = 0.95; Table 3). In contrast, the average probability of being available for capture was equal between the sexes at Site I (Pr[*γ*_m,C_ > *γ*_f,C_] = 0.45; Table 3).

## 4 DISCUSSION

Integrating models of distinct but related demographic processes can help extract more information from CMR data that are labor‐intensive to collect. Growth and survival are fundamental processes that interact to determine the age and size structure of a population, and therefore are natural choices to integrate into one model. For our focal species, jointly analyzing growth and survival provided clear benefits. Given the low recapture rates of San Francisco gartersnakes, we could not measure the SVL of most individuals in the majority of years following their initial capture. Still, based on the importance of size to the demography of vertebrates (Sauer & Slade, 1987a) and for survival in snakes in particular (Brown & Weatherhead, 1999; Hyslop et al., 2012; Rose, Wylie et al., 2018), it was desirable to account for snake size when modeling survival. Embedding a growth model into our CMR model allowed us to impute snake SVL when it was not observed, while ensuring that uncertainty in this state variable was propagated when estimating relationships between size and survival.

Although there was substantial uncertainty in annual survival for the smallest and largest snakes (due to small sample sizes and less precise estimates of availability for capture), there was a pattern of nearly equal survival for snakes between 300 and 550 mm SVL, and a decrease in survival for female snakes as SVL increased from 550 mm to 700 mm. Whether survival further decreased or plateaued for the largest San Francisco gartersnakes was unclear due to our small sample of snakes >700 mm SVL. Reduced survival for larger female San Francisco gartersnakes could be a sign of senescence (Reinke et al., 2020) or the survival costs of reproduction for large females (Luiselli et al., 1996; Madsen & Shine, 1993). Previous studies have documented costs of reproduction in female natricine snakes such as reduced locomotor ability (Seigel et al., 1987) and lower post‐partum body condition (Brown & Weatherhead, 1997). Actuarial senescence (a decrease in survival with age) has been documented for colubrid snakes (Cayuela et al., 2020) including some populations of the western terrestrial gartersnake (*T*. *elegans*; Colchero et al., 2019; Miller et al., 2011). The strength of actuarial senescence can depend on where a population falls on the fast–slow continuum of life history, with greater senescence expected in populations with fast life histories (Cayuela et al., 2020).

Although the San Francisco gartersnake has a restricted range in coastal northern California, our results hint that populations might differ in how each sex balances the needs for growth and survival to sexual maturity. Within our study populations, there were a few differences in patterns of growth and survival of female and male snakes. The finding that females reached greater lengths than males in each population is in keeping with other studies of San Francisco gartersnakes (Barry, 1994; Reeder et al., 2015). Males reached larger asymptotic length at Site I than at Site C, but females had similar asymptotic lengths at all five sites. Females exhibited higher annual survival than males at Site C, and males at Site C approached their asymptotic length at a faster rate than females. At Site I, the two sexes had nearly equal rates of growth, with some support for males having higher survival than females. We observed more spatial than temporal variation in snake growth rates, indicating that sites explain more of the variation in growth than annual fluctuations in the environment. In contrast, for survival, there were greater differences between the two sexes within a site than between mean estimates at each site. Data on reproductive traits from multiple populations could help elucidate the degree of life‐history variation in San Francisco gartersnakes. It is possible that differences in climate between coastal and inland populations could be one factor in determining life‐history strategies of San Francisco gartersnake populations. Daily temperature maxima and the influence of marine layer‐induced fog can differ between inland and coastal sites on the San Francisco Peninsula (Torregrosa et al., 2016). Prey availability has also been identified as a cause of life‐history variation among populations of the western terrestrial gartersnake (Bronikowski & Arnold, 1999), but we lacked quantitative data on the abundance and composition of prey at our sites.

Gartersnakes have been the subject of much research into life‐history strategies and trade‐offs between reproduction, growth, and survival. Substantial variation in life‐history traits existed among populations of the common gartersnake (*T*. *sirtalis*) in Canada, with females from eastern populations reaching sexual maturity at a smaller size and producing larger litters of smaller neonates compared with western populations (Gregory & Larsen, 1993, 1996). At a finer scale, nearby populations of the western terrestrial gartersnake in northeastern California, USA, exhibit contrasting life histories depending on their habitat. Western terrestrial gartersnakes inhabiting cooler mountain meadows with more variable prey grow more slowly, produce smaller litters, and have higher annual survival than fast‐growing, early maturing, highly fecund snakes inhabiting lakeshore areas with abundant prey (Bronikowski & Arnold, 1999). Life‐history variation is not purely of scientific interest; optimal management and conservation actions can depend on life‐history characteristics. The elasticity of population growth to changes in vital rates varied between ecotypes of western terrestrial gartersnakes (Miller et al., 2011). Potential differences in life history among populations of San Francisco gartersnakes could have implications for the conservation of this endangered subspecies.

Estimating size‐dependent survival is valuable for informing conservation actions for imperiled species. For example, if conservation groups had a goal to reintroduce San Francisco gartersnakes to suitable habitat, which individuals are the best targets for translocation to reintroduce populations? Annual survival rates and how survival varies with size could influence which life stages to translocate and the number of translocated individuals necessary to establish a viable population. Another important consideration for population viability is environmental stochasticity. For example, survival rates of San Francisco gartersnakes fluctuated from year to year, which could lead to large interannual variation in the abundance of snakes within a population. A previous study at Site C from 2008 to 2010 found high annual survival rates for this species, with mean estimates between 0.74 and 0.88 (depending on the model used; Halstead et al., 2011). The longer time series in this study shows that those two years had the highest survival rates from 2007 to 2020. Thus, results from a short‐term CMR study could give overly optimistic or pessimistic projections of population viability if the survival rates fall at the high or low end of the distribution observed through time. Projecting the viability of populations under environmental stochasticity requires estimates of the variance in vital rates in space and time (Caswell, 2001; Rees & Ellner, 2009). Future demographic models for San Francisco gartersnakes that account for spatiotemporal variation in growth and survival will more accurately capture the dynamics of natural populations.

Snake size not only affected annual survival but it was also positively related to recapture probability and availability on‐site for capture. Failing to account for the relationship between individual size and recapture probability could lead to an inaccurate estimate of how size and survival are related. For example, funnel traps can be size‐selective such that both small and large snakes have lower capture probability than intermediate sizes (Rose, Wylie et al., 2018; Willson et al., 2008). If capture probability declines with size for the largest snakes, the lower recapture rate could be incorrectly attributed to a lower survival rate for large snakes, if the size–capture relationship was ignored or was assumed to follow a linear relationship. By using penalized splines to model size–capture probability and size–survival relationships, we could estimate nonlinear curves that fit the data with fewer assumptions. Although there is high uncertainty in the spline functions for the smallest and largest individuals, the fitted curve accurately reflects what can be concluded from the data. In contrast, fitting parametric functions could result in overly precise credible intervals induced by a fixed functional shape.

The flexibility of using splines in a CMR model comes at a cost of needing to estimate additional parameters (e.g., compared with an intercept and slope in a linear function), and potentially greater uncertainty in the response variable where data are sparse. Overall, our recapture rates of San Francisco gartersnakes were low, with only 18% of snakes recaptured in at least one year following their initial capture and marking. This led to high uncertainty in our survival estimates, particularly for the youngest, smallest snakes and the largest snakes, which were rarely recaptured. For elusive snake species, complex statistical models alone cannot resolve the issue of low capture rates (Steen, 2010). Increasing recapture probabilities compared with the current study could require increased spatial coverage of trap arrays within a site (to reduce the effect of temporary emigration) and a longer duration of sampling within each year. For example, Reeder et al. (2015) sampled a population of San Francisco gartersnakes with a much greater density of trap arrays and for a longer duration each year (>70 days) than in the current study, and were able to estimate abundance of snakes >300 mm SVL with good precision. Still, increased sampling effort (i.e., increased trap density) alone cannot overcome low recapture rates for individuals of certain life stages if the sampling method exhibits bias in the sizes captured (Rose, Wylie et al., 2018; Willson et al., 2008), or if the sampling period is not aligned seasonally with the availability of certain life stages (e.g., if sampling takes place before parturition and therefore neonates are unavailable).

Reducing uncertainty in our estimates of survival for the smallest and largest San Francisco gartersnakes could require a combination of increased sampling effort, greater duration of sampling within a year, and complementary approaches to studying the demography of these snakes. For reptiles, early life stages are notoriously difficult to capture and recapture (Pike et al., 2008). Estimating survival rates for small, young snakes could require integrating fecundity data along with CMR into an integrated population model (Schaub & Abadi, 2011). Likewise, estimates of availability of San Francisco gartersnakes for capture exhibited large uncertainty. The use of spatial capture–recapture models that account for snake movement (Ergon & Gardner, 2014) could improve estimates of availability for capture and lead to more precise estimates of survival. Unfortunately, within‐year captures of San Francisco gartersnakes in our data were too sparse to fit spatial capture–recapture models and estimate the locations of individuals’ activity centers. With increased sampling effort at a site, it might be possible to obtain enough within‐year recaptures to fit spatial capture–recapture models, at least for snakes in the size range best sampled with funnel traps. Another potentially fruitful approach might be to use radiotelemetry of large adult females, in conjunction with survival analysis (Williams et al., 2002), to precisely estimate annual survival rates of this life stage.

### 4.1 Summary and conclusions

Our study is an important step for developing a robust predictive framework for San Francisco gartersnake population dynamics. Our results have shown that individual size influences growth and survival rates in this endangered subspecies and that growth and survival vary among populations and over time. The growth and survival parameters estimated here, in conjunction with fecundity data, could inform a size‐structured demographic model that would identify size ranges of San Francisco gartersnakes to focus conservation efforts on to increase population viability. An important next step would be to link observed individual, interpopulation, and temporal variation in growth and survival to environmental variables and land management practices. Researchers with long‐term CMR data could benefit from integrating models of growth, survival, and other demographic processes to reveal insights into how individual covariates affect vital rates and ultimately population growth.

## CONFLICT OF INTEREST

We have no conflict of interest to disclose.

## AUTHOR CONTRIBUTION

**Jonathan Rose:** Conceptualization (equal); Data curation (equal); Formal analysis (lead); Methodology (equal); Software (lead); Writing – original draft (lead); Writing – review & editing (equal). **Richard Kim:** Data curation (equal); Formal analysis (supporting); Investigation (equal); Methodology (equal); Software (supporting); Writing – original draft (supporting); Writing – review & editing (equal). **Elliot J Schoenig:** Conceptualization (supporting); Data curation (equal); Investigation (equal); Writing – review & editing (supporting). **Patrick C Lien:** Conceptualization (supporting); Data curation (equal); Investigation (equal); Writing – review & editing (supporting). **Brian J. Halstead:** Conceptualization (equal); Formal analysis (supporting); Funding acquisition (lead); Methodology (equal); Project administration (lead); Supervision (lead); Writing – review & editing (equal).

## Supporting information

Fig S1

Fig S2

Fig S3

Fig S4

Fig S5

Fig S6

Supplementary Material

## Data Availability

The data used in this study are archived on the U.S. Geological Survey's ScienceBase (https://doi.org/10.5066/P9SC36I8), and code necessary to reproduce our growth and survival analyses are available on the USGS WERC GitLab (https://doi.org/10.5066/P9700BBK).
