# Supplementary figures and images for: Integrating growth and survival models for flexible estimation of size‐dependent survival in a cryptic, endangered snake
Source: Ecol Evol. 2022 Apr 6;12(4):e8799. doi: 10.1002/ece3.8799 (PMC8987119; doi:10.1002/ece3.8799)

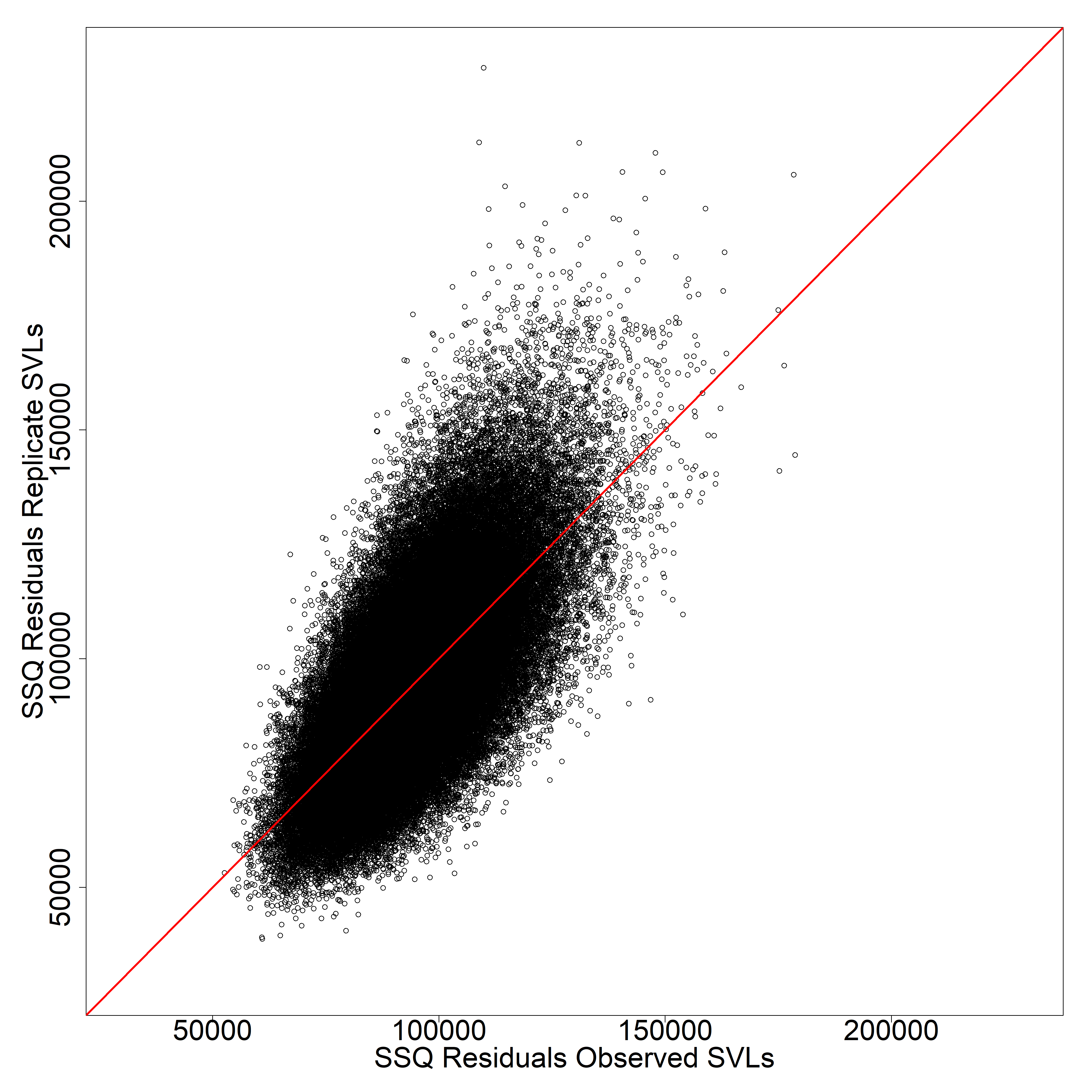

Supplement: Supplementary file 1 — Fig S1 [file ECE3-12-e8799-s002.tiff]

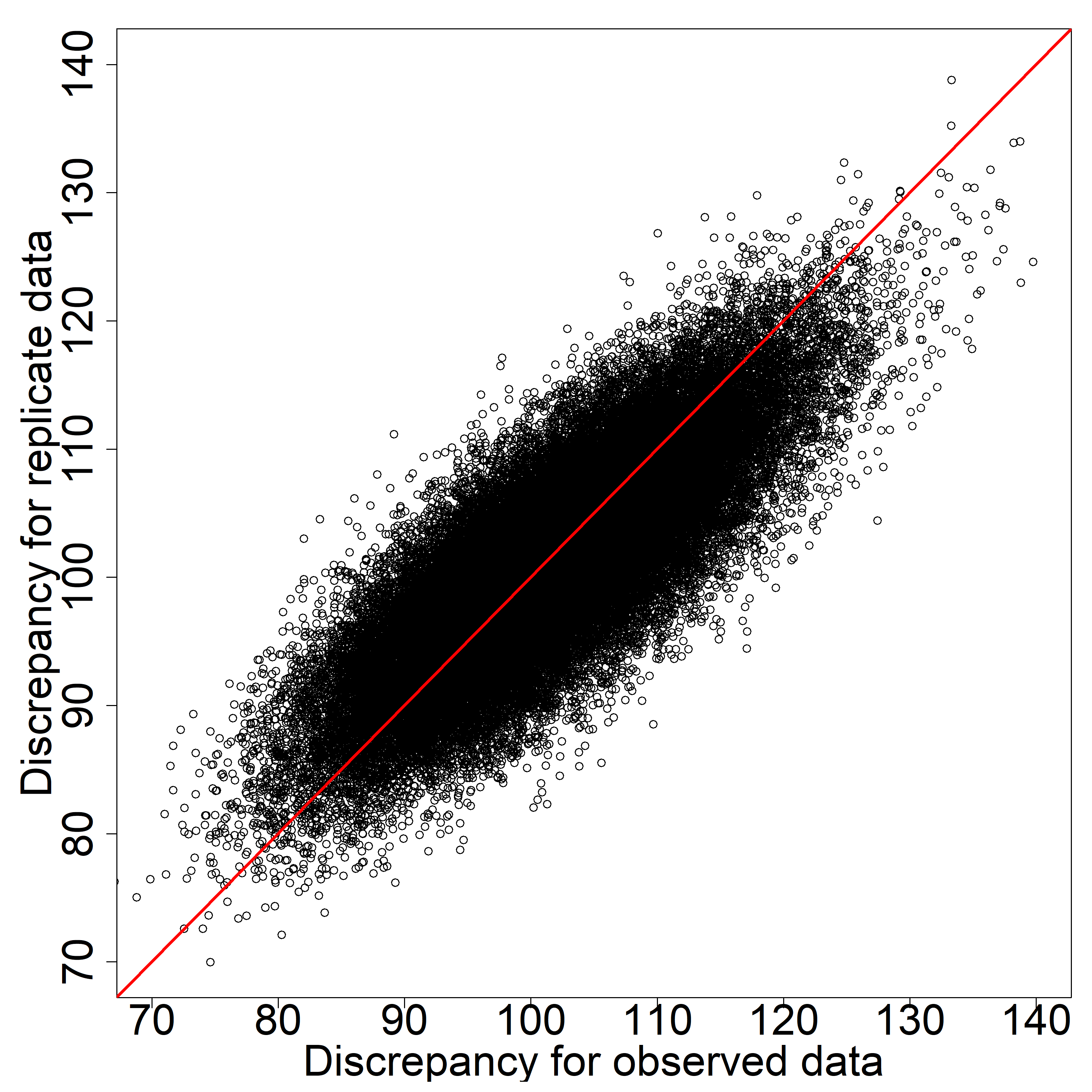

Supplement: Supplementary file 2 — Fig S2 [file ECE3-12-e8799-s007.tiff]

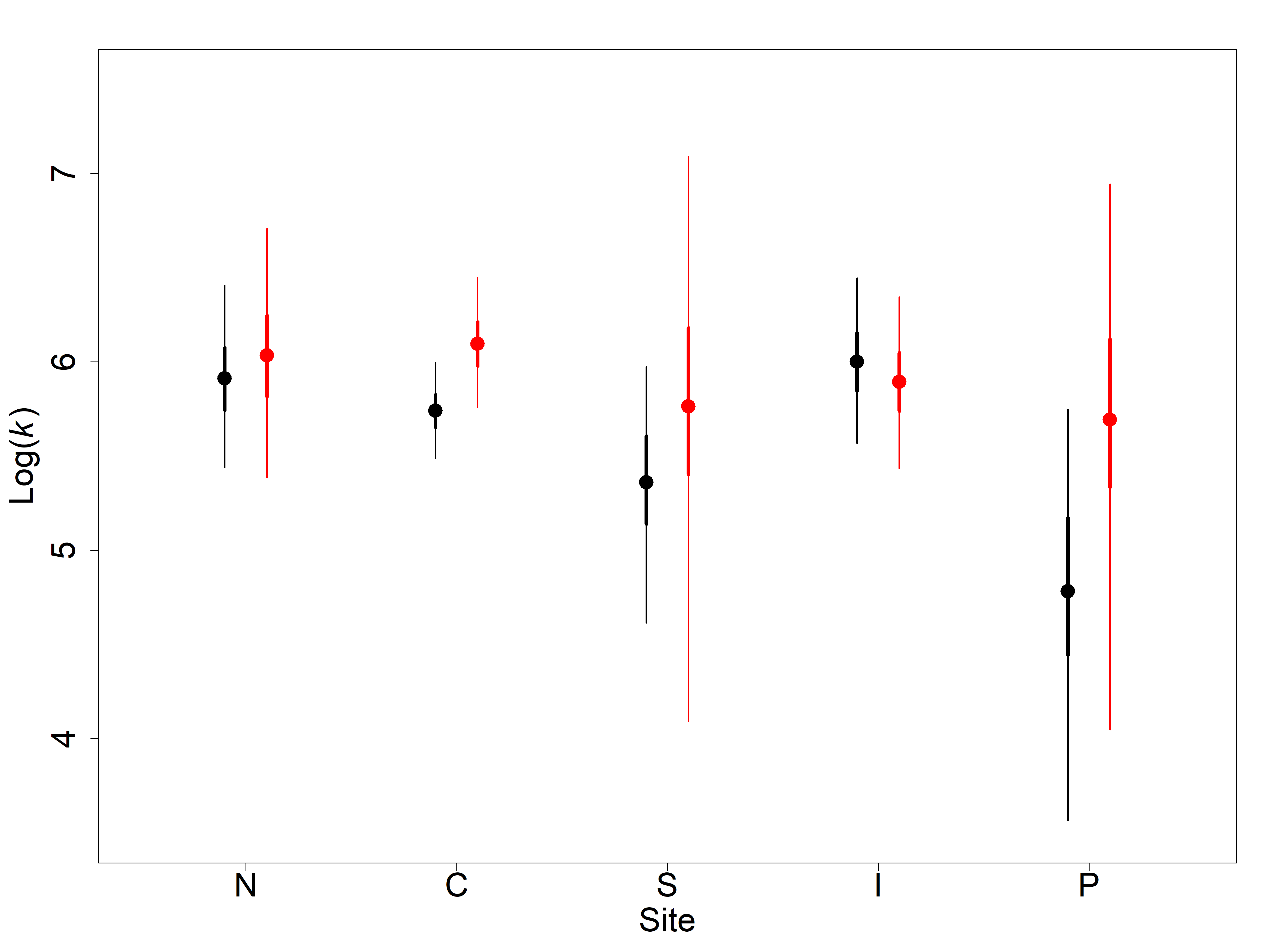

Supplement: Supplementary file 3 — Fig S3 [file ECE3-12-e8799-s006.tiff]

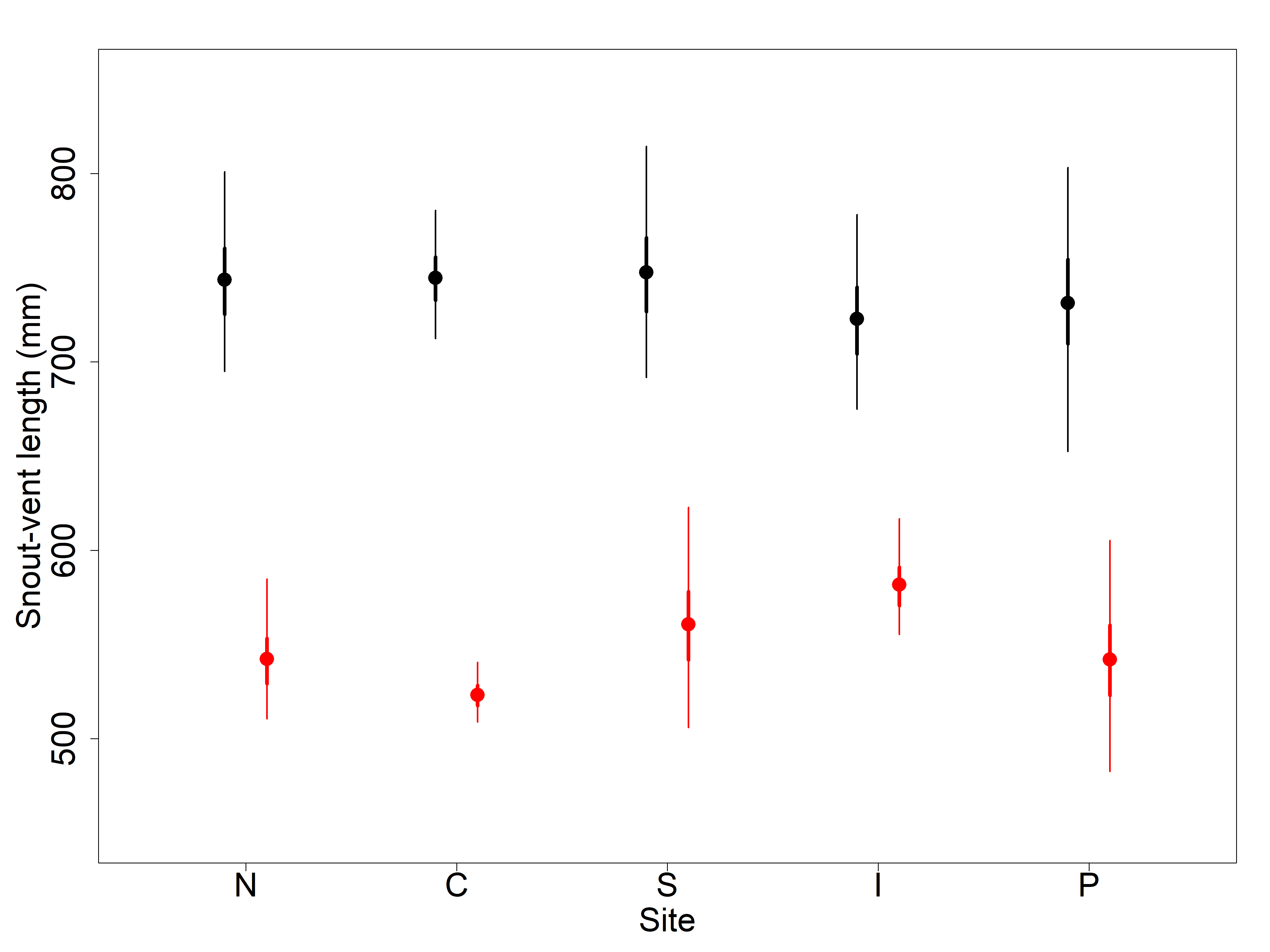

Supplement: Supplementary file 4 — Fig S4 [file ECE3-12-e8799-s001.tiff]

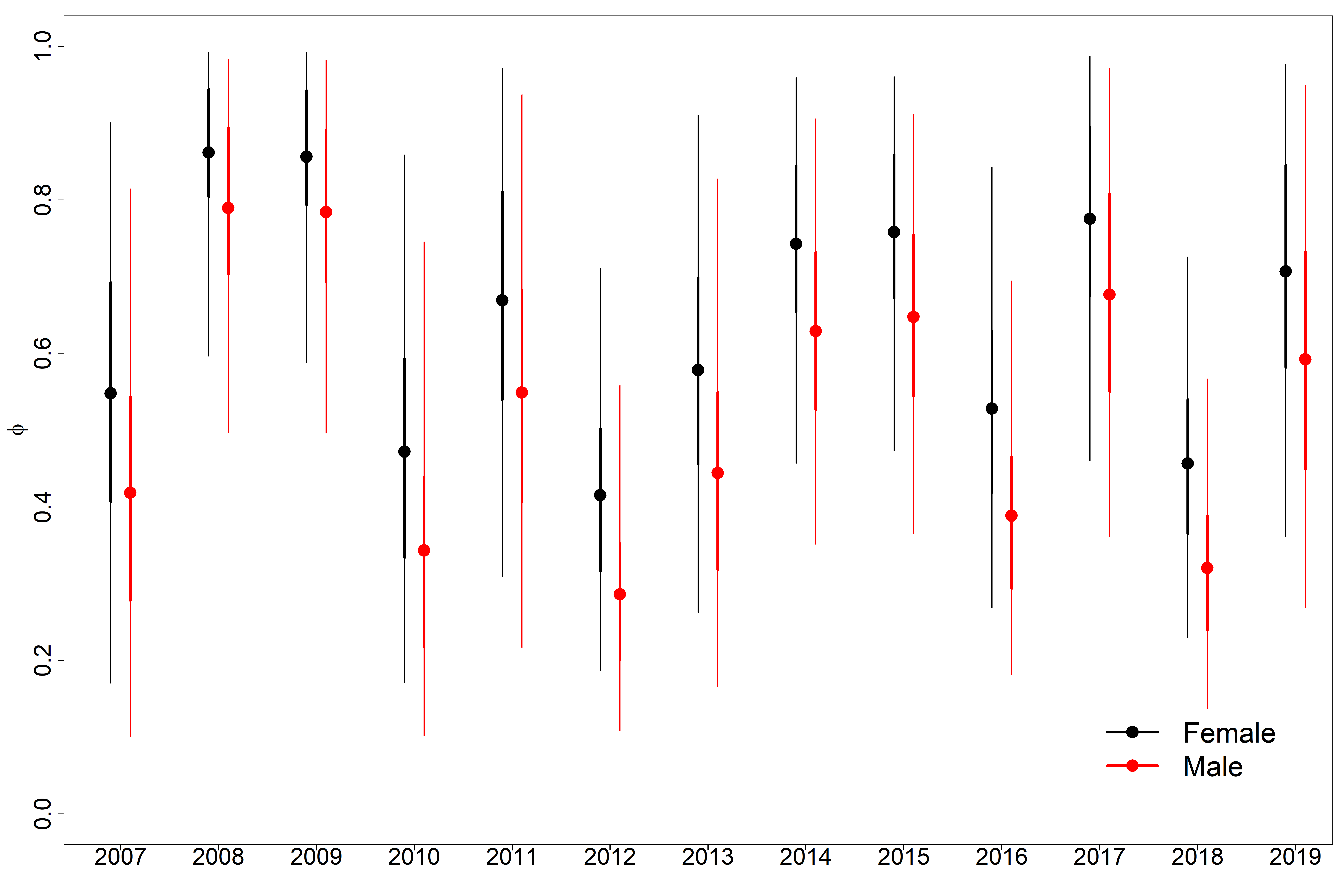

Supplement: Supplementary file 5 — Fig S5 [file ECE3-12-e8799-s005.tiff]

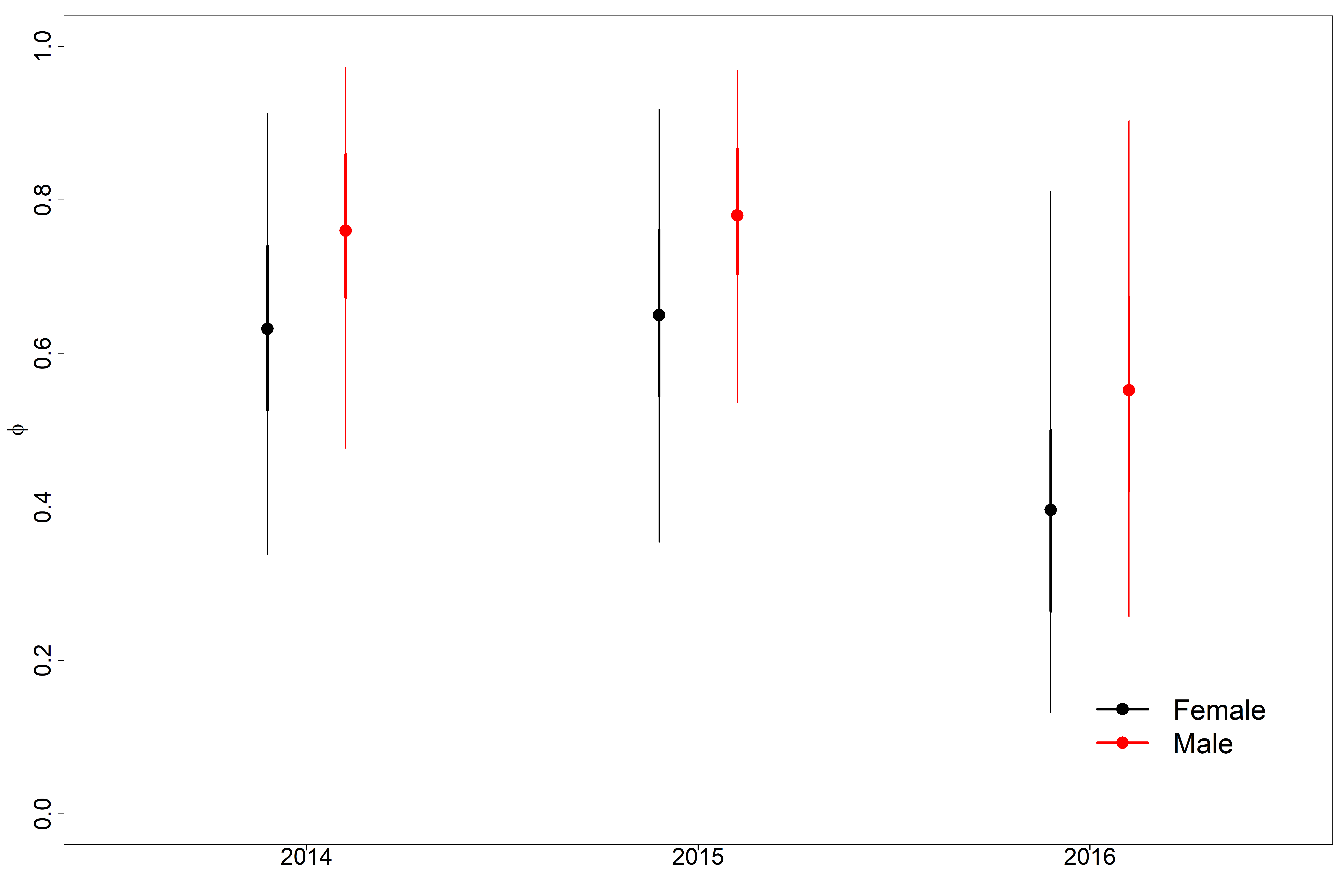

Supplement: Supplementary file 6 — Fig S6 [file ECE3-12-e8799-s003.tiff]
