# Supplementary material for: Integrating growth and survival models for flexible estimation of size‐dependent survival in a cryptic, endangered snake
Source: Ecol Evol. 2022 Apr 6;12(4):e8799. doi: 10.1002/ece3.8799 (PMC8987119; doi:10.1002/ece3.8799)
Supplement: Supplementary file 7 — Supplementary Material [file ECE3-12-e8799-s004.docx]

**Appendix S1.** Additional methods and results for “Integrating growth and survival models for flexible estimation of size-dependent survival in a cryptic, endangered snake”.

**Methods**

We sampled for San Francisco gartersnakes (*Thamnophis sirtalis tetrataenia*) at five sites for periods ranging from two years (sites N, P, and S) to 4 years (site I) and 14 years (Site C). Sites were primarily sampled using drift fences with funnel traps (hereinafter: trap-arrays) but artificial objects were also used at sites C and I. The number of trap-arrays used per site was constant over time, but the number of cover boards deployed varied between years at Site C (Table S1). Trap-arrays were set in close proximity to wetland habitat or in upland habitat nearby to wetlands. Trap-arrays were within an average distance of 60 m from wetlands, but the distance from wetland edge varied by site and trap-array (Table S1). Trap-arrays were generally closer to wetland habitat at Site S and Site P, whereas the distance from wetland was greater for some trap-arrays at Site C. The sampling design varied among sites based on the original objective of the study at a given site. Sites N, S, and P were sampled in 2018 and 2019 as part of a study on the genetic effective population size and estimated abundance (i.e., census size) of San Francisco gartersnakes (Wood et al., 2020). Site I was sampled each year from 2014 to 2017 with the objective of studying the demography of San Francisco gartersnakes as well as their distribution in wetland and upland habitat on a former cattle ranch (Kim et al., 2018). Site C has been sampled every year since 2007 as part of a long-term study on the demography of San Francisco gartersnakes on an open-space preserve. The location of trap-arrays at Site C was were chosen as part of a stratified-random design to cover both upland and wetland habitat, resulting in a greater average distance between trap-arrays and the edge of wetlands (Halstead et al., 2011).

We estimated the effective area sampled at each site by buffering the location of drift-fence and funnel trap-arrays by 200 m. We selected a 200 m buffer based on the fact that 95% of inter-trap movements by San Francisco gartersnakes at sites N, P, S, and C in 2018 were < 200 m (Wood et al. 2020). We clipped the buffered area to remove non-habitat such as suburban development, beaches, and ocean from the effective area sampled. We then used observations from the field in conjunction with satellite imagery from ArcGIS version 10.8.1 and outlines of water bodies from the National Hydrography Dataset (U.S. Geological Survey, 2019) to estimate the area of wetland habitat within the effective area sampled at each site. We calculated the area of upland habitat as the remaining habitat within the effective sampled area that was not wetland habitat (Table S2). The density of trap-arrays was highest at Site P, given the limited habitat available at this site. Trap-array density was intermediate and comparable between sites S and N, and the density of trap-arrays was comparably lower for sites I and C, which included larger patches of upland habitat between wetlands (Table S2).

*Environmental covariates*

We collected air temperature at 1 m above ground at the time of sampling (checking traps and cover objects) for most sample days. For days in which field-collected air temperatures were missing, we imputed the air temperature based on the relationship between field-collected air temperature and mean daily air temperature from the gridMET dataset (Abatzoglou, 2013). We embedded a linear regression relating the field air temperature (AT*_f_*) to mean daily air temperature from gridMET (*AT_g_*) using a site-specific intercept (*c*_s_) and slope (*h*_s_) into the robust-design CJS model (Eq. S1). The gridMET mean air temperature was used to predict the expected field air temperature (v*_AT,f_*). The true, unobserved field air temperature was then modeled using a normal distribution centered on the expected field air temperature, with a standard deviation (*σ_AT,f_*) based on residual variation in field air temperatures.

(S1) $v= c_{s}+h_{s}*{AT}_{g}$

(S2) ${AT}_{F} \sim N\left( v_{AT,f},\sigma_{AT,f} \right)$

*Covariance between growth and survival*

Vital rates are often assumed to be independent in demographic studies, but survival, growth, and reproduction in a given year can be correlated (Hindle et al., 2018). To account for potential correlation among annual growth and survival rates, we drew annual random effects on survival (*ϕ*) and growth (*k*) from a multi-variate normal (MVN) distribution (Eq. S3).

(S3) $B_{\phi,k,t} \sim MVN\left( 0,\Sigma\right)$

The multi-variate normal distribution has a covariance matrix, Σ (Eq. S4), with the variance of the annual survival random effect (*σ*^2^_ϕ,t_), the variance of the annual random effect on *k* (*σ*^2^_k,t_), and the correlation between the two parameters (*ρ*). We defined each individual parameter of the covariance matrix, using the methods of Riecke et al. (2019). We placed a Uniform(minimum=-1, maximum=1) prior on the correlation parameter, ρ, and Exponential (rate=1) priors on the standard deviation of the annual random effects on *ϕ* (σ _ϕ,t_) and *k* (σ*_k_*_,t_).

(S4) $\Sigma= \left[ \begin{matrix} \sigma_{\phi,t}^{2} & \sigma_{\phi,t}\sigma_{k,t}\rho\\ \sigma_{\phi,t}\sigma_{k,t}\rho& \sigma_{k,t}^{2} \end{matrix} \right]$

**References**

Abatzoglou, J.T., 2013. Development of gridded surface meteorological data for ecological applications and modelling. Int. J. Climatol. 33, 121–131. https://doi.org/10.1002/joc.3413

Halstead, B.J., Wylie, G.D., Amarello, M., Smith, J.J., Thompson, M.E., Routman, E.J., Casazza, M.L., 2011. Demography of the San Francisco Gartersnake in Coastal San Mateo County, California. J. Fish Wildl. Manag. 2, 41–48. https://doi.org/10.3996/012011-JFWM-009

Hindle, B.J., Rees, M., Sheppard, A.W., Quintana-Ascencio, P.F., Menges, E.S., Childs, D.Z., 2018. Exploring population responses to environmental change when there is never enough data: a factor analytic approach. Methods Ecol. Evol. 9, 2283–2293. https://doi.org/10.1111/2041-210X.13085

Kim, R., Halstead, B.J., Wylie, G.D., Casazza, M.L., 2018. Distribution and Demography of San Francisco Gartersnakes (*Thamnophis sirtalis tetrataenia*) at Mindego Ranch, Russian Ridge Open Space Preserve, San Mateo County, California. U.S. Geological Survey Open-File Report 2018-1063, 80 p., https://doi.org/10.3133/ofr20181063

Riecke, T. V., Sedinger, B.S., Williams, P.J., Leach, A.G., Sedinger, J.S., 2019. Estimating correlations among demographic parameters in population models. Ecol. Evol. 9, 13521–13531. https://doi.org/10.1002/ece3.5809

U.S. Geological Survey, 2019. National Hydrography Dataset [WWW Document]. URL https://www.usgs.gov/core-science-systems/ngp/national-hydrography/access-national-hydrography-products (accessed 4.10.20).

Wood, D.A., Rose, J.P., Halstead, B.J., Stoelting, R.E., Swaim, K.E., Vandergast, A.G., 2020. Combining genetic and demographic monitoring better informs conservation of an endangered urban snake. PLoS One 15, e0231744. https://doi.org/10.1371/journal.pone.0231744

**Table S1.** Summary of annual sampling effort at each site during the study period: numbers of sampling days, trap-arrays (drift fence with four funnel traps), and cover objects.

| Year | Site | Sampling days | Trap-arrays deployed | Cover objects deployed | Start date | End date |
| --- | --- | --- | --- | --- | --- | --- |
| 2007 | C | 58 | 24 | 224 | 4/5/2022 | 8/7/2007 |
| 2008 | C | 40 | 24 | 162 | 3/26/2008 | 6/12/2008 |
| 2009 | C | 63 | 24 | 189 | 3/18/2009 | 9/3/2009 |
| 2010 | C | 60 | 24 | 96 | 4/28/2010 | 6/26/2010 |
| 2011 | C | 21 | 24 | 27 | 6/11/2011 | 6/30/2011 |
| 2012 | C | 62 | 24 | 99 | 3/21/2012 | 6/11/2012 |
| 2013 | C | 48 | 24 | 110 | 4/2/2022 | 7/21/2013 |
| 2014 | C | 46 | 24 | 75 | 4/5/2014 | 5/20/2014 |
| 2015 | C | 64 | 24 | 121 | 2/26/2015 | 9/18/2015 |
| 2016 | C | 53 | 24 | 108 | 4/1/2016 | 5/23/2022 |
| 2017 | C | 54 | 24 | 84 | 3/9/2017 | 5/24/2017 |
| 2018 | C | 51 | 24 | 101 | 4/2/2018 | 5/24/2018 |
| 2019 | C | 54 | 24 | 101 | 3/30/2019 | 5/28/2019 |
| 2020 | C | 46 | 24 | 101 | 4/1/2020 | 5/26/2020 |
| 2014 | I | 48 | 12 | 131 | 4/5/2014 | 5/22/2014 |
| 2015 | I | 64 | 12 | 131 | 2/26/2015 | 9/18/2015 |
| 2016 | I | 54 | 12 | 131 | 4/1/2016 | 5/24/2022 |
| 2017 | I | 47 | 12 | 131 | 3/9/2017 | 5/19/2017 |
| 2018 | N | 45 | 12 | 0 | 4/5/2018 | 5/21/2018 |
| 2019 | N | 41 | 12 | 0 | 4/8/2019 | 5/23/2019 |
| 2018 | P | 36 | 12 | 0 | 4/19/2018 | 5/24/2018 |
| 2019 | P | 30 | 12 | 0 | 4/19/2019 | 5/24/2019 |
| 2018 | S | 33 | 12 | 0 | 5/7/2018 | 6/8/2018 |
| 2019 | S | 40 | 12 | 0 | 4/10/2019 | 5/23/2019 |

**Table S2.** Effective area sampled and its habitat components (wetland vs. upland), trap-array density, and distance trap-arrays were located from wetland habitat for each of the five study sites. Distance from wetlands is the mean distance (in meters) that trap-arrays were located from a wetland edge at each site, followed by the range in parentheses). Effective area sampled was calculated by creating a 200 m buffer around all trap and cover objects at a site, assuming that most movements by San Francisco gartersnakes are < 200 m (Wood et al., 2020), and removing non-habitat such as suburban development and coastline.

| Site | Effective area sampled (km^2^) | Wetland habitat area (km^2^) | Upland habitat area (km^2^) | Trap-arrays | Arrays/km^2^ | Distance from wetlands |
| --- | --- | --- | --- | --- | --- | --- |
| N | 0.53 | 0.05 | 0.48 | 12 | 22.6 | 40 (1 - 86) |
| C | 1.82 | 0.12 | 1.70 | 24 | 13.2 | 60 (3 - 215) |
| S | 0.41 | 0.06 | 0.35 | 12 | 29.3 | 5 (1 - 16) |
| I | 0.98 | 0.03 | 0.95 | 12 | 12.2 | 42 (2 - 89) |
| P | 0.2 | 0.03 | 0.17 | 12 | 60.0 | 15 (1 - 40) |

**Table S3.** Parameter symbols and descriptions for von Bertalanffy growth and robust-design capture-mark-recapture models. The column JAGS parameter column lists the corresponding name of the parameter in the JAGS code used to fit the model.

| **Equation** | **Function** | **Symbol** | **Parameter description** | **JAGS parameter** |
| --- | --- | --- | --- | --- |
| 1 | von Bertalanffy growth | EL | Expected SVL of individual i at time t | EL |
| 1 | von Bertalanffy growth | *a* | Asymptotic length (SVL) | a |
| 1 | von Bertalanffy growth | L | measured length (SVL) | L |
| 1 | von Bertalanffy growth | *k* | von Bertalanffy growth coefficient | k |
|  |  |  |  | - |
| 2 | von Bertalanffy growth | *L* | Observed SVL of individual i at time t | L |
| 2 | von Bertalanffy growth | ε | Random effect of size at time t | sd.eps/tau.eps |
|  |  |  |  |  |
| 3 | Linear predictor for *k* | µ_k,f_ | Mean value of log(k) for a female snake | mu.k.f |
| 3 | Linear predictor for *k* | α_sex,s_ | Sex and site-specific random effect on *k* | alpha.k.sex.site |
| 3 | Linear predictor for *k* | ζ | Random effect of year on *k* | zeta.k.t |
| 3 | Linear predictor for *k* | ι | Individual random effect on *k* | iota.k.ind |
| 3 | Linear predictor for *k* | β | Effect of male sex on *k* | beta.k.m |
|  |  |  |  |  |
| 4 | Linear predictor for *a* | υ | Mean value of *a* for a female snake | ups.a.f |
| 4 | Linear predictor for *a* | η | Sex and site-specific random effect on *a* | eta.a.sex.site |
| 4 | Linear predictor for *a* | θ | Effect of male sex on *a* | theta.a.m |
|  |  |  |  |  |
| 5 | Linear predictor for ϕ | ϕ | Apparent survival | phi |
| 5 | Linear predictor for ϕ | π | Intercept of ϕ on logit scale | pi.phi |
| 5 | Linear predictor for ϕ | x | Snout-vent length (standardized) | svl.std |
| 5 | Linear predictor for ϕ | *f*_ϕ_(x) | Spline function of size on ϕ | tp_phi |
| 5 | Linear predictor for ϕ | 𝜉 | Annual random effect on ϕ | B[t,1] |
|  |  |  |  |  |
| 6 | Linear predictor for *p* | *p* | Daily recapture probability | *p* |
| 6 | Linear predictor for *p* | ο | Intercept of *p* on logit scale | omi.p |
| 6 | Linear predictor for *p* | x | Snout-vent length (standardized) | svl.std |
| 6 | Linear predictor for *p* | δ | Vector of coefficients for covariate effects on *p* | delta.vec.p |
| 6 | Linear predictor for *p* | ***W*** | Array of covariates on *p* | W.array |
| 6 | Linear predictor for *p* | *f_p_*(x) | Spline function of size on *p* | tp_p |
| 6 | Linear predictor for *p* | ω | Site- and year-specific random effect on *p* | omega.p.j |
|  |  |  |  |  |
| 7 | Linear predictor for γ | γ | Availability for recapture (present on-site) | gamma |
| 7 | Linear predictor for γ | ς | Intercept of γ on logit scale | fsig.gamma |
| 7 | Linear predictor for γ | λ | Effect of snout-vent length on γ | lambda.gamma |
| 7 | Linear predictor for γ | x | Snout-vent length (standardized) | svl.std |
| 7 | Linear predictor for γ | 𝜈 | Site- and year-specific random effect on γ | nu.gamma |
|  |  |  |  |  |
|  |  |  |  |  |
| 8 | Spline effect on *p* or ϕ | q | Fixed effect of SVL on response | q_p and q_phi |
| 8 | Spline effect on *p* or ϕ | b | Random effects coefficients for spline | b_p and b_phi |
| 8 | Spline effect on *p* or ϕ | κ | Fixed knots along range of observed SVL values | - |
| 8 | Spline effect on *p* or ϕ | G | Number of knots in the spline | G |
| 8 | Spline effect on *p* or ϕ | d | Degree of the spline | - |
|  |  |  |  |  |
| 9 | Prior for b parameters | b | Random effects coefficients for effect of SVL | b_p and b_phi |
| 9 | Prior for b parameters | σ*_b_* | Standard deviation of distribution for b | sigma.b.p and sigma.b.phi |
|  |  |  |  |  |
| S1 | Linear predictor for air temp | v*_AT,f_* | Expected field air temperature | v.at |
| S1 | Linear predictor for air temp | *c_s_* | Intercept for relationship between *AT_f_* and *AT_g_* | c.at |
| S1 | Linear predictor for air temp | *h_s_* | Slope of relationship between *AT_f_* and *AT_g_* | h.at.grid |
| S1 | Linear predictor for air temp | *AT_g_* | Modeled air temperature from gridMET | grid.at |
|  |  |  |  |  |
| S2 | Model for inferring air temp | *AT_f_* | Observed field air temperature | W.array[*s*,*t*,*j*,1] |
| S2 | Model for inferring air temp | v*_AT,f_* | Expected field air temperature | v.at |
| S2 | Model for inferring air temp | σ*_AT,f_* | Standard deviation of residual variation in AT | sd.at.grid |
|  |  |  |  |  |
| S3 | Covariance among *k* and ϕ | B_ϕ_*_,k,t_* | Annual random effects on survival (ϕ) and growth (*k*) | B |
| S3 | Covariance among *k* and ϕ | Σ | Variance-covariance matrix for annual random effects | VCOV |
|  |  |  |  |  |
| S4 | Covariance matrix | Σ | Variance-covariance matrix for annual random effects | VCOV |
| S4 | Covariance matrix | ρ | Correlation between annual random effects on *k* and ϕ | rho |
| S4 | Covariance matrix | σ*_k_*_,_*_t_* | Standard deviation of annual random effect on *k* | sd.k.t |
| S4 | Covariance matrix | σ_ϕ,_*_t_* | Standard deviation of annual random effect on ϕ | sd.phi.t |

**Supplemental Figures**

**
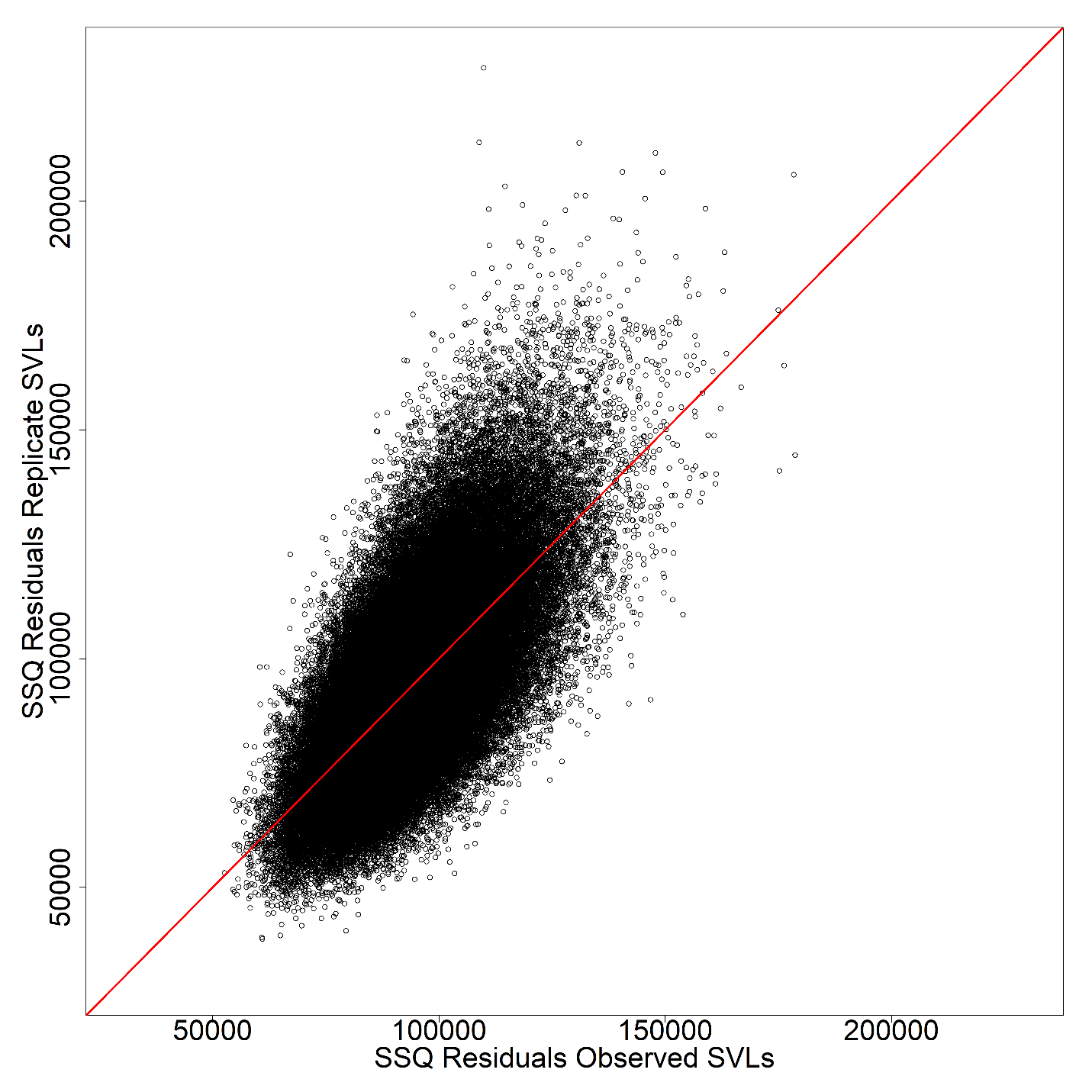
**

**Figure S1.** Goodness-of-fit using a posterior predictive check for the von Bertalanffy growth model for San Francisco gartersnakes (*Thamnophis sirtalis tetrataenia*). The x-axis is the sum-of-squares (SSQ) of residuals comparing observed SVL values to expected SVL predicted by the growth model. The y-axis is the SSQ of residuals comparing replicate SVL values generated by the model to expected SVL predicted by the growth model. Each point represents one MCMC sample from the growth model. The red line is a 1:1 line with intercept=0 and slope=1.


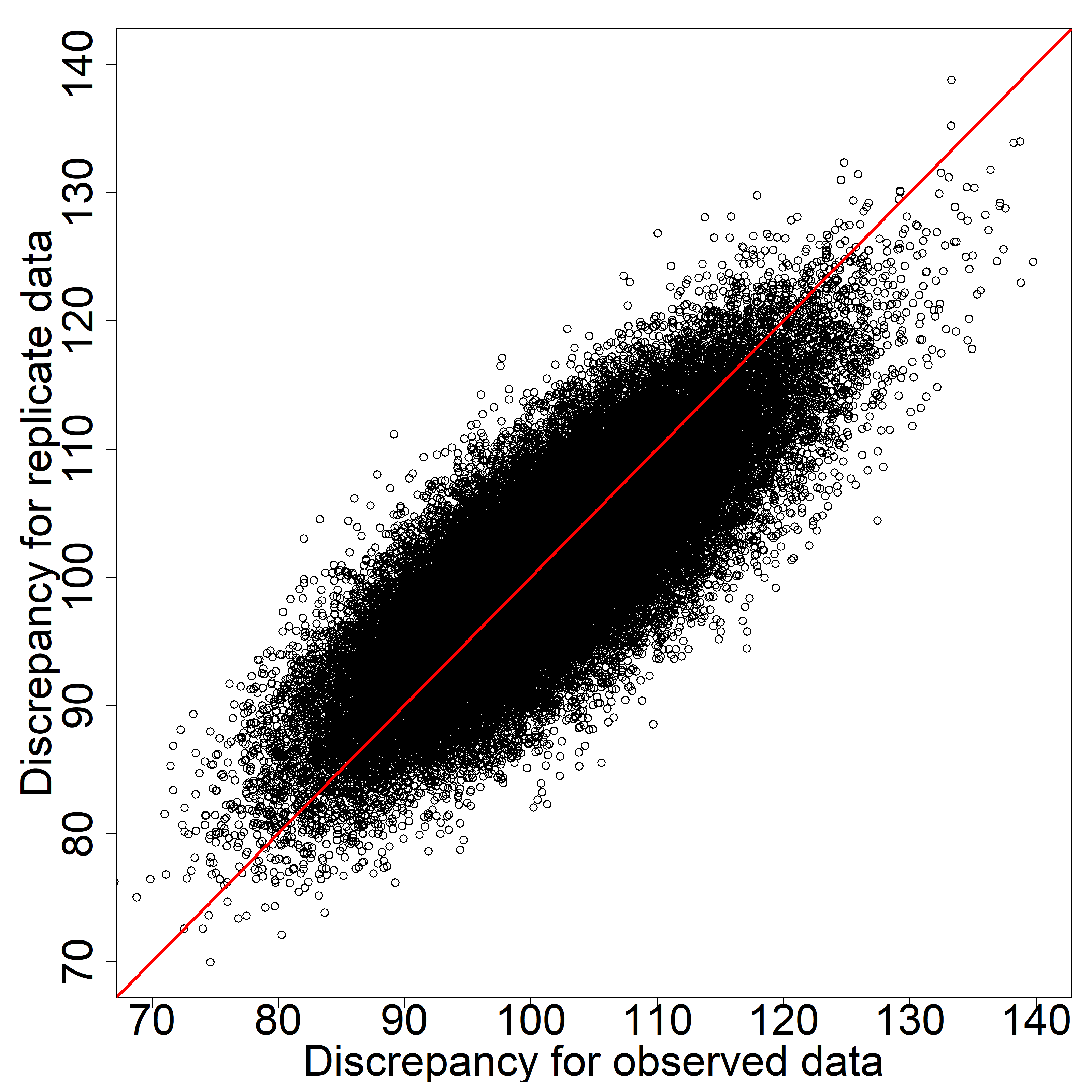


**Figure S2.** Goodness-of-fit using a posterior predictive check for the robust-design model for San Francisco gartersnakes (*Thamnophis sirtalis tetrataenia*). The x-axis is the sum of the Freeman-Tukey statistic comparing observed number of recaptures to the expected number of recaptures predicted by the CJS model. The y-axis is the sum of the Freeman-Tukey statistic comparing replicate number of recaptures generated by the model to the expected number of recaptures predicted by the CJS model. Each point represents one MCMC sample from the CJS model. The red line is a 1:1 line with intercept=0 and slope=1.


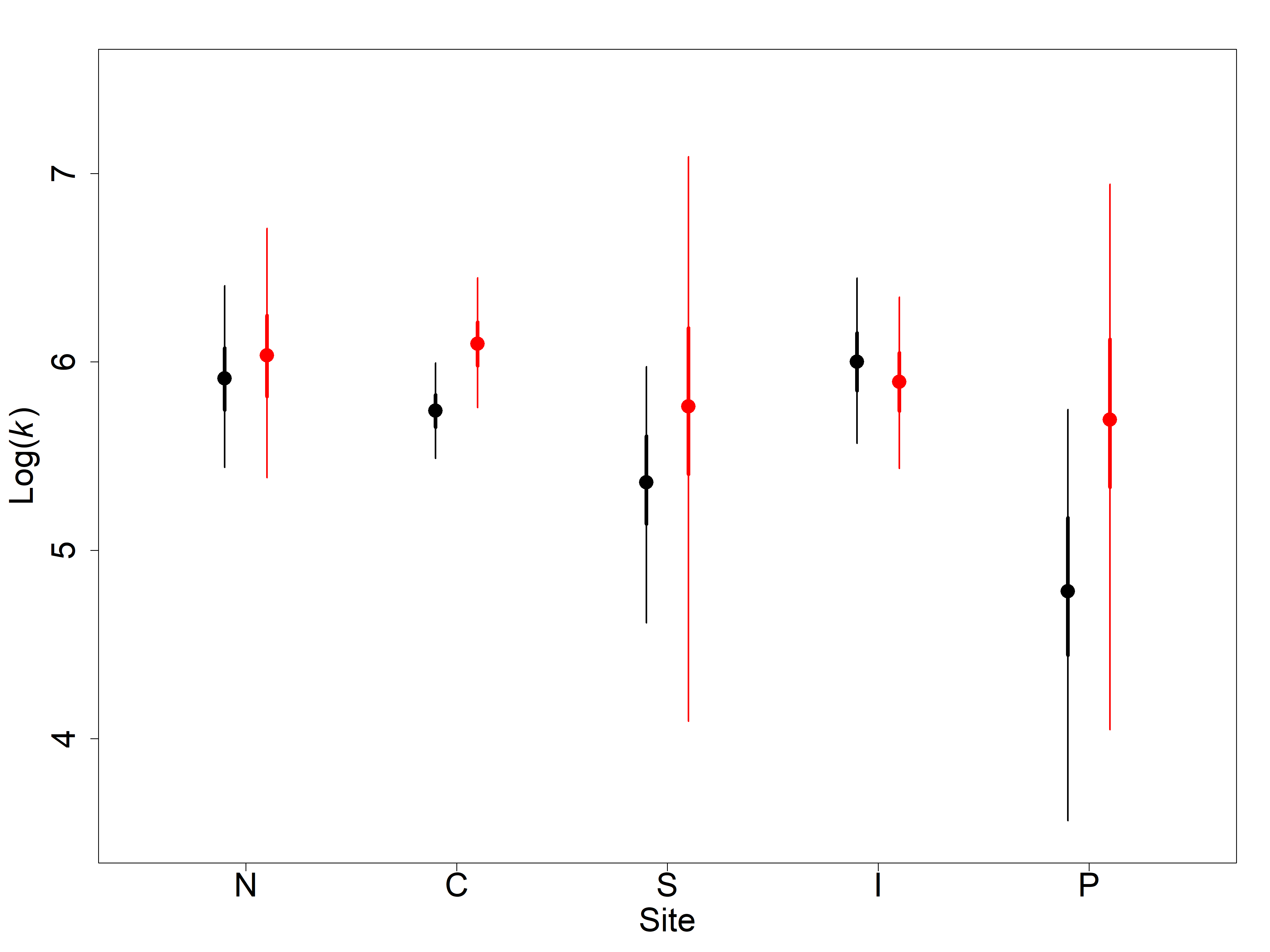


**Figure S3.** Site- and sex-specific estimates of log(*k*), the log of the growth coefficient from the von Bertalanffy growth model, for San Francisco gartersnakes (*Thamnophis sirtalis tetrataenia*). Black lines and points are estimates for female snakes, red lines and points are estimates for male snakes. Points represent means of the posterior distribution, thick lines are 50% credible intervals, and thin lines are 95% credible intervals.

**
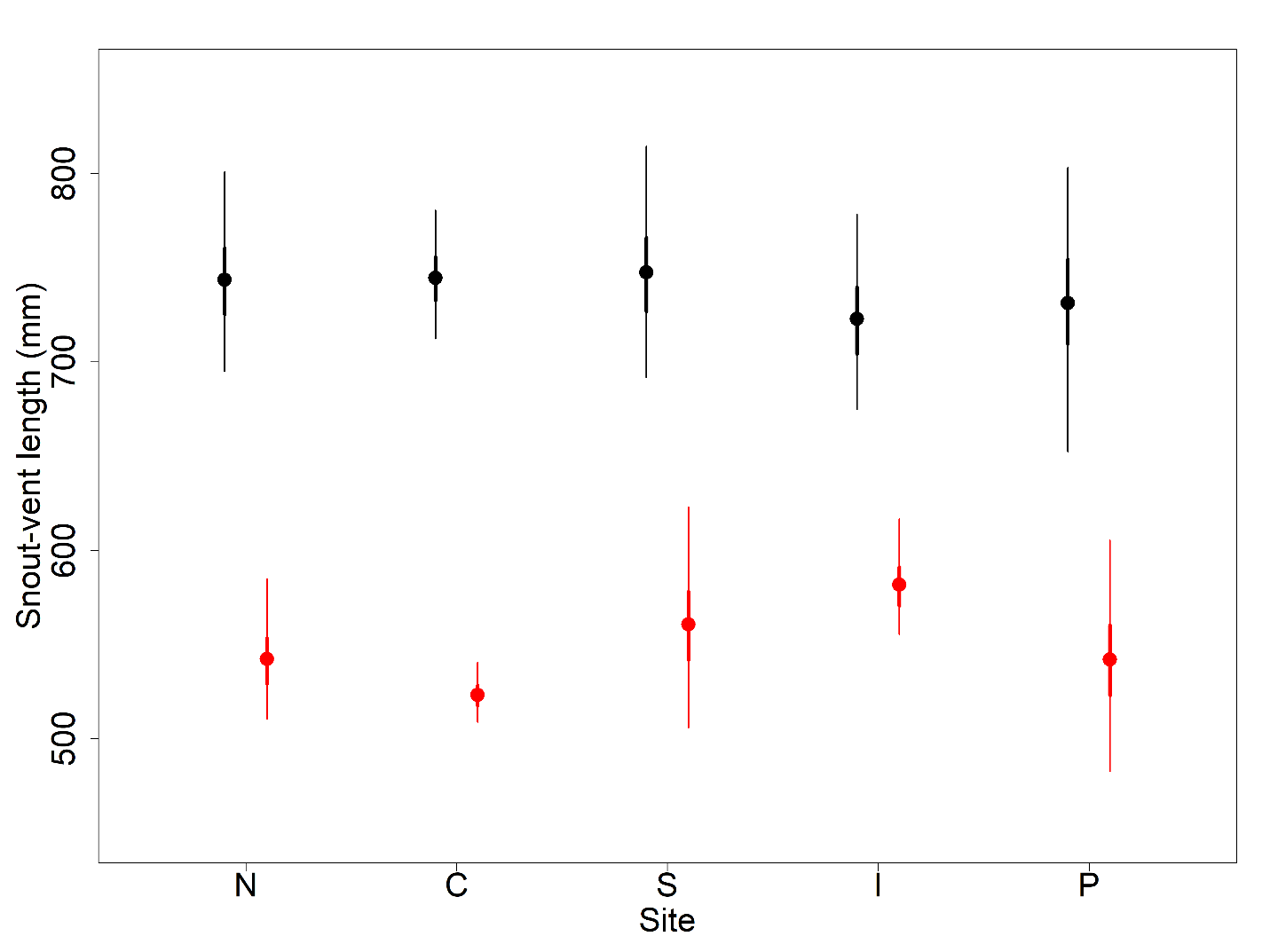
**

**Figure S4.** Site- and sex-specific estimates of *a*, the asymptotic snout-vent length (SVL) in mm from the von Bertalanffy growth model, for San Francisco gartersnakes (*Thamnophis sirtalis tetrataenia*). Black lines and points are estimates for female snakes, red lines and points are estimates for male snakes. Points represent means of the posterior distribution, thick lines are 50% credible intervals, and thin lines are 95% credible intervals.

**
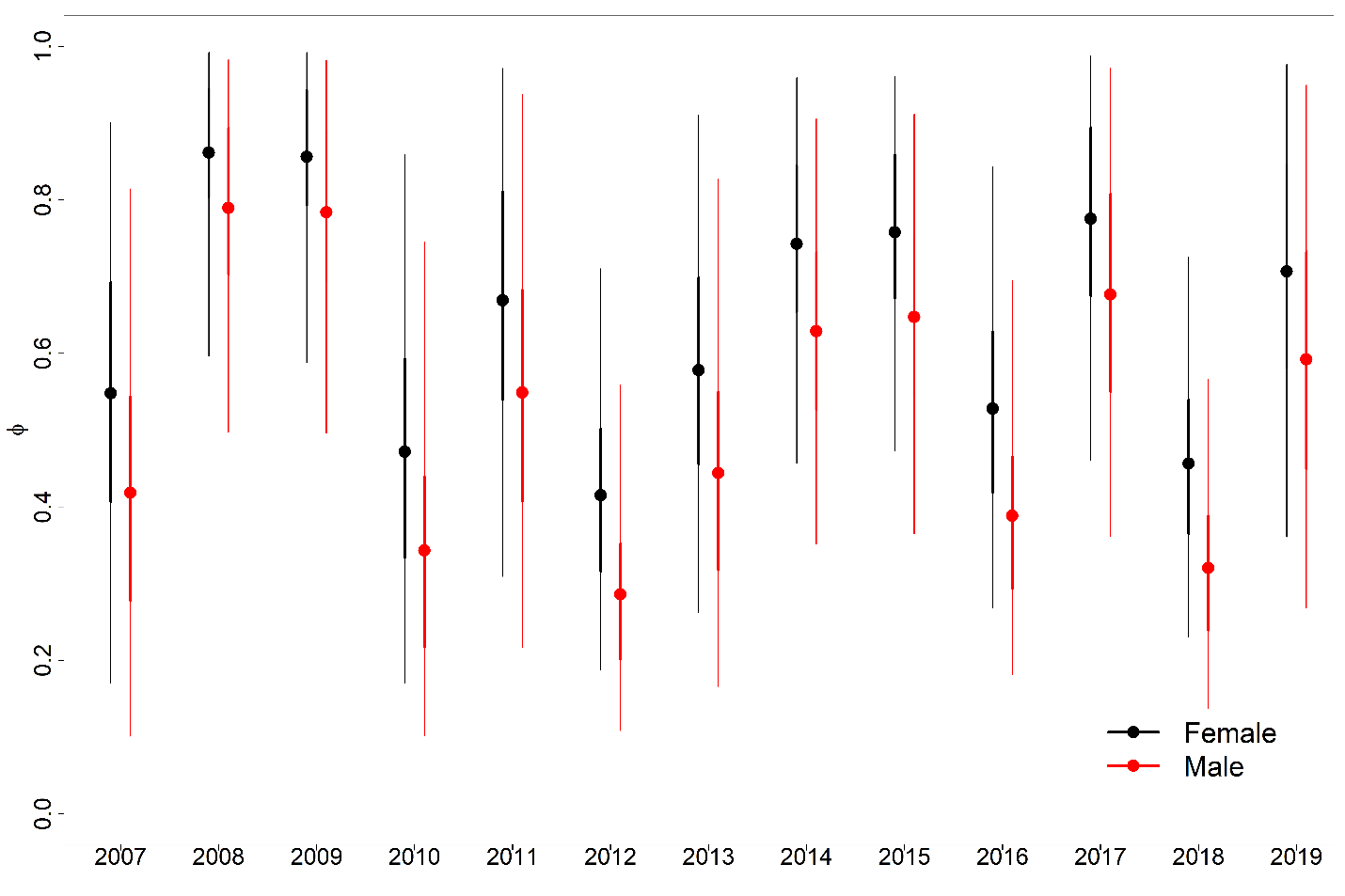
**

**Figure S5.** Annual estimates of apparent survival (ϕ) for San Francisco gartersnakes (*Thamnophis sirtalis tetrataenia*) at Site C from 2007–2020. Values on the x-axis represent the first year of the survival interval (e.g., 2007 represents survival from 2007 to 2008). Black lines and points are estimates for female snakes, red lines and points are estimates for male snakes. Points represent means of the posterior distribution, thick lines are 50% credible intervals, and thin lines are 95% credible intervals.


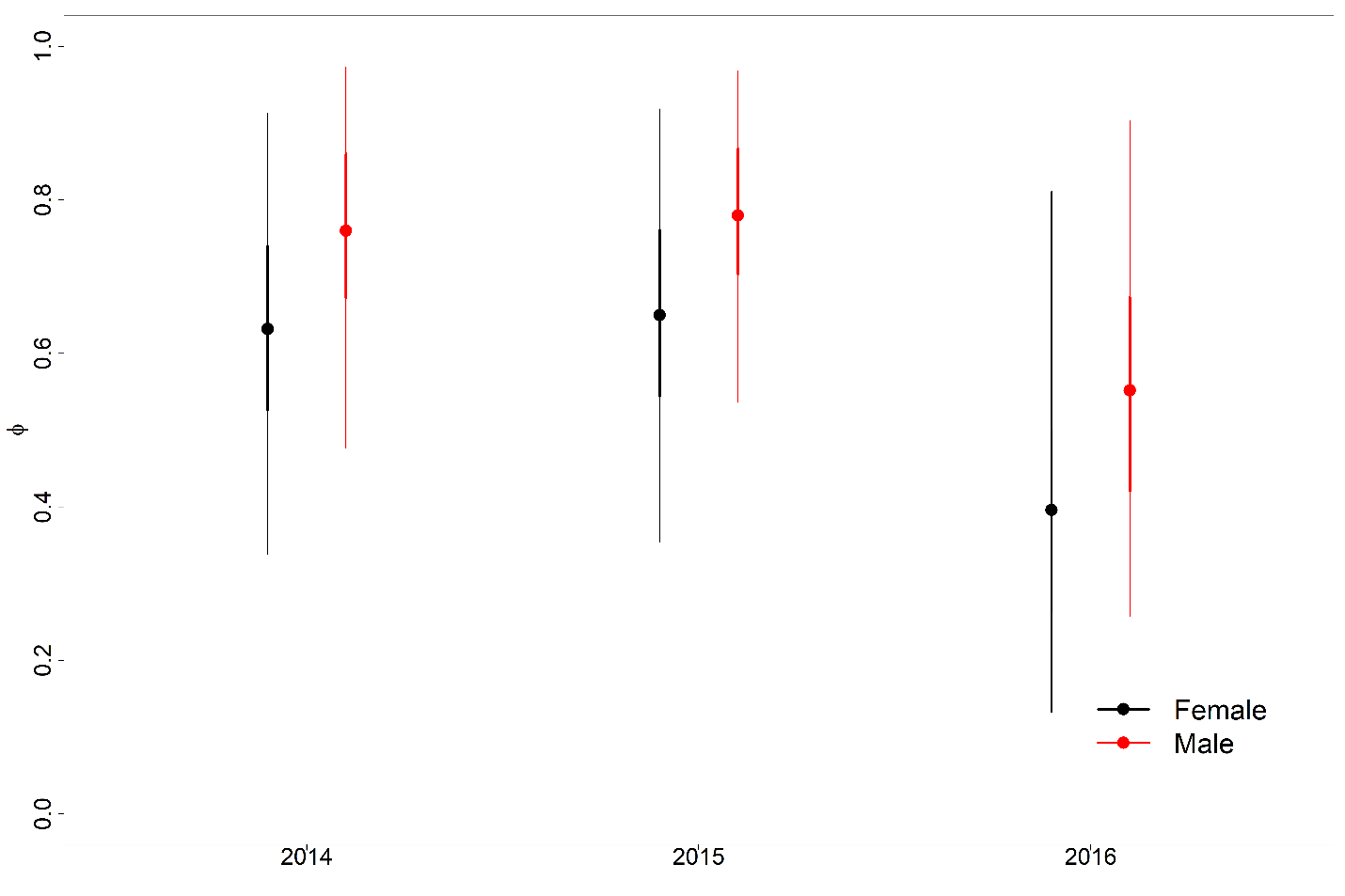


**Figure S6.** Annual estimates of apparent survival (ϕ) for San Francisco gartersnakes (*Thamnophis sirtalis tetrataenia*) at Site I from 2014–2017. Values on the x-axis represent the first year of the survival interval (e.g., 2014 represents survival from 2014 to 2015). Black lines and points are estimates for female snakes, red lines and points are estimates for male snakes. Points represent means of the posterior distribution, thick lines are 50% credible intervals, and thin lines are 95% credible intervals.
